# FlgN Is Required for Flagellum-Based Motility by Bacillus subtilis

**DOI:** 10.1128/JB.01599-14

**Published:** 2014-06

**Authors:** Lynne S. Cairns, Victoria L. Marlow, Taryn B. Kiley, Christopher Birchall, Adam Ostrowski, Phillip D. Aldridge, Nicola R. Stanley-Wall

**Affiliations:** a Division of Molecular Microbiology, College of Life Sciences, University of Dundee, Dundee, United Kingdom; b Centre for Bacterial Cell Biology, Newcastle University, Newcastle upon Tyne, United Kingdom

## Abstract

The assembly of the bacterial flagellum is exquisitely controlled. Flagellar biosynthesis is underpinned by a specialized type III secretion system that allows export of proteins from the cytoplasm to the nascent structure. Bacillus subtilis regulates flagellar assembly using both conserved and species-specific mechanisms. Here, we show that YvyG is essential for flagellar filament assembly. We define YvyG as an orthologue of the Salmonella enterica serovar Typhimurium type III secretion system chaperone, FlgN, which is required for the export of the hook-filament junction proteins, FlgK and FlgL. Deletion of *flgN* (*yvyG*) results in a nonmotile phenotype that is attributable to a decrease in *hag* translation and a complete lack of filament polymerization. Analyses indicate that a *flgK-flgL* double mutant strain phenocopies deletion of *flgN* and that overexpression of *flgK-flgL* cannot complement the motility defect of a Δ*flgN* strain. Furthermore, in contrast to previous work suggesting that phosphorylation of FlgN alters its subcellular localization, we show that mutation of the identified tyrosine and arginine FlgN phosphorylation sites has no effect on motility. These data emphasize that flagellar biosynthesis is differentially regulated in B. subtilis from classically studied Gram-negative flagellar systems and questions the biological relevance of some posttranslational modifications identified by global proteomic approaches.

## INTRODUCTION

The bacterial flagellum is a complex molecular motor that has been shown to play roles in motility, surface adherence, biofilm structure, and signal transduction (1–4). The flagellum is organized into three main structural components: the basal body, hook, and filament (1). The basal body consists of the flagellar motor, which is required to power rotation of the flagellum, and a type III secretion (T3S) system that permits the export of proteins required for the biosynthesis of the hook and filament. The hook is a flexible joint that permits a change in the angle of rotation of the flagellum, while the filament acts as a propeller to drive movement. Biosynthesis of the flagellum is tightly regulated at the level of transcription. In the Gram-positive bacterium Bacillus subtilis, the proteins needed for the hook-basal body (HBB) are transcribed in the 31-gene *fla-che* operon (5, 6). The penultimate gene of this operon, *sigD*, encodes the sigma factor σ^D^ (7, 8) that activates transcription of the late flagellar genes: the flagellar filament gene *hag*; the flagellar stator genes *motA* and *motB*; the anti-sigma factor *flgM*; the hook-filament junction genes *flgK* and *flgL* (9); and the autolysins (10). In wild-type B. subtilis, while all cells transcribe the *fla-che* operon, only a subpopulation of the cells synthesize flagella (11, 12). This is due to heterogeneity in *sigD* transcription such that a threshold level of *sigD* transcription must be reached to allow sufficient σ^D^ protein to accumulate and activate σ^D^-regulated promoters (11). The net result is that transcription is temporally ordered such that the HBB genes are expressed before the filament genes (1).

As well as being controlled at the level of transcription, flagellar biosynthesis is regulated posttranscriptionally by flagellar type III secretion system (T3S) chaperones. Regulation at this level has been described most extensively in Salmonella enterica serovar Typhimurium, with little known about the function of chaperones in Gram-positive bacterial species (13, 14). T3S chaperones are small proteins that bind their cognate substrate(s) in the cytoplasm, protecting the substrate from degradation and/or preventing aggregation (15, 16). Chaperones therefore allow the efficient transport of the substrate to the export machinery. Chaperone-substrate complexes reach the secretion apparatus with the aid of the soluble export apparatus proteins FliI and FliH (17), while empty chaperones are recycled with the aid of FliJ (18). Following interaction of the chaperone-substrate complex with the C-terminal cytoplasmic domain of the integral membrane protein FlhA, a series of protein-protein interactions facilitates the entry of the substrate protein to the export gate (19–21), and its subsequent secretion is driven by proton motive force (22, 23). In *S*. Typhimurium, FliS is a specific chaperone for flagellin (24), FliT is specific for the FliD filament cap protein, and FlgN (*S*T-FlgN) is specific for the hook-filament junction proteins, FlgK and FlgL (25, 26). Recently, it has been shown that FliS is required for Hag (flagellin) secretion in B. subtilis (13, 14). In addition, *in silico* analysis has suggested that YvyG of B. subtilis is an orthologue of the *S*. Typhimurium protein FlgN (27). However, a defined function for YvyG has not yet been determined experimentally.

Several global proteomic screens have been conducted using B. subtilis with the goal of examining the extent and diversity of posttranslational modification (28–30). Intriguingly, these experiments identified YvyG as being phosphorylated on tyrosine 49 (29) and arginine 60 (30). Posttranslational modification of proteins can control cell fate in several ways: (i) by altering protein localization and half-life (31), (ii) by controlling protein activity and affinity to ligands (32), and (iii) by the disruption or promotion of protein-protein interactions (33). The B. subtilis flagellum has recently been shown to be regulated by mechanisms not identified in other bacterial species (34, 35). Therefore, given the potential for YvyG to play a crucial role in the tightly controlled process of flagellar biosynthesis (31, 36), we hypothesized that protein phosphorylation might present an additional route for B. subtilis to regulate flagellar assembly and, therefore, motility. Thus, we aimed to define the function of YvyG during motility by B. subtilis and to ascertain the *in vivo* role of YvyG tyrosine and arginine phosphorylation.

Work presented here identifies B. subtilis YvyG (here referred to as B. subtilis FlgN [*Bs*-FlgN]) as an orthologue of the *S*. Typhimurium T3S chaperone FlgN, as previously suggested by *in silico* analysis (27). Consistent with this, we prove for the first time that FlgN is required for both swimming and swarming motility in B. subtilis. The lack of motility in the B. subtilis *flgN* deletion strain is linked to a block in flagellar biosynthesis. This is a consequence of a complete lack of filament assembly. Analysis of a Δ*flgK-flgL* double mutant strain demonstrates that the strain phenocopies the *flgN* mutation. Collectively, these data prove that in B. subtilis FlgN is required for flagellar assembly, perhaps by acting as a chaperone for FlgK and FlgL. In *S*. Typhimurium, overexpression of *flgK* can compensate for the motility defect of a Δ*flgN* strain (16). However, data presented here demonstrates that in B. subtilis deletion of *flgN* cannot be compensated for by the overexpression of *flgK* and *flgL*. This leads to the conclusion that there is a stricter dependence on the presence of FlgN in B. subtilis for motility than there is for FlgN in *S*. Typhimurium (16, 36). Finally, through the use of site-directed mutagenesis, we demonstrate that mutation of the tyrosine and arginine phosphorylation sites of FlgN has no effect on the ability of B. subtilis to become motile. In summary, these data emphasize that flagellar biosynthesis is differentially regulated in B. subtilis in comparison to the classically studied Gram-negative bacteria and additionally raises questions regarding the biological relevance of some posttranslational modifications identified by global proteomic approaches.

## MATERIALS AND METHODS

### Growth conditions and strain construction.

Escherichia coli and Bacillus subtilis strains were routinely grown in Luria-Bertani (LB) medium (10 g liter^−1^ NaCl, 5 g liter^−1^ yeast extract, 10 g liter^−1^ tryptone) or on LB plates supplemented with 1.5% agar at 37°C unless otherwise stated. E. coli strain MC1061 [F′ *lacI*^q^ *lacZ*ΔM15 Tn*10*(*tet*)] was used for the routine construction and maintenance of plasmids. When required, antibiotics were used at the following concentrations: 100 μg ml^−1^ ampicillin, 100 μg ml^−1^ spectinomycin, 25 μg ml^−1^ chloramphenicol, 10 μg ml^−1^ kanamycin (B. subtilis), 50 μg ml^−1^ kanamycin (E. coli), 1 μg ml^−1^ erythromycin, and 25 μg ml^−1^ lincomycin. Strains were constructed using standard protocols. Phage transductions were carried out as previously described (37). When appropriate, isopropyl β-d-1-thiogalactopyranoside (IPTG) was added at the concentrations indicated in the figures or figure legends. A full list of strains used in this study is provided in Table S1 in the supplemental material.

### Construction of in-frame deletion strains.

To construct the in-frame deletion of *flgN*, an approach similar to that previously described was used (38). The upstream region of *flgN* was amplified from genomic DNA using primers NSW938 and NSW939, purified, and digested with XbaI and SalI using the restriction sites engineered into the primers. The downstream region of *flgN* was amplified using primers NSW936 and NSW937, purified, and digested with BamHI and XbaI using the restriction sites engineered into the primers. The fragments were simultaneously ligated into pUC19 and sequenced, prior to introduction into pMAD (39), to produce plasmid pNW399. Strain NRS3570 (NCIB3610 Δ*flgN*) was generated by integration and curing of the region contained in pNW399 in strain NCIB3610. Strains NRS4041 (NCIB3610 Δ*fliD*), NRS4042 (NCIB3610 Δ*flgE*), and NRS4060 (NCIB3610 Δ*flgK-flgL*) were constructed in a similar manner using the primers and plasmids detailed in Tables S2 and S3 in the supplemental material, respectively, and in the supplemental methods.

### Introduction of site-specific mutations to the chromosome.

To introduce site-specific mutations to the chromosome of NCIB3610, a similar approach to that previously described was used (39). Plasmids pNW801 and pNW1012 were used to introduce site-specific mutations in codon 49 of *flgN*. Primers NSW936 and NSW939 were used to amplify a 1,725-bp region of DNA containing the complete *flgN* coding region. The PCR fragment was cloned into pUC19 using SalI and SphI restriction sites engineered into the primers, resulting in plasmid pNW398. Primer sets NSW942 and NSW943 (Y^49^A) and NSW1436 and NSW1437 (Y^49^E) were used to introduce point mutations to pNW398 using site-directed mutagenesis by the QuikChange method, according to the manufacturer's instructions (Stratagene). The resulting plasmids were sequenced to ensure that the correct mutations were introduced. The mutated *flgN* coding region was excised and cloned into pMAD for integration into NCIB3610, as described for the construction of in-frame deletion strains. Further plasmids were constructed in an identical manner for *hag* (Hag T^209^C) and *flgN* (with the R-to-A or R-to-E mutation at position 60 encoded by *flgN* [*flgN*-R^60^A or *flgN*-R^60^E, respectively]). Full details of the plasmids and primers used are provided in Tables S2 and S3, respectively, in the supplemental material.

### Sigma A antibodies.

To overexpress and purify the σ^A^ protein from B. subtilis for antibody preparation, the *sigA* gene was amplified from the chromosome of the strain NCIB3610 using primers NSW860 and NSW861. The NcoI and XhoI sites engineered into the primer sequence are underlined in Table S3 in the supplemental material. The resulting PCR product was digested with NcoI and XhoI and cloned into the expression vector pEHISGFPTEV (40) to yield a His_6_-green fluorescent protein (GFP)-σ^A^ fusion construct named pNW642. A tobacco etch virus (TEV) protease recognition site was placed between the *gfp* and *sigA* coding regions.

E. coli BL21(DE3) cells carrying the pNW642 vector were grown in LB broth containing ampicillin to an optical density at 600 nm (OD_600_) of 0.3 at 37°C. The cells were chilled to 20°C, and protein expression was induced with 50 μM IPTG overnight. Cells were collected by centrifugation and resuspended in lysis buffer (25 mM Tris [pH 7.5], 250 mM NaCl, 5 mM dithiothreitol [DTT], 30 mM imidazole, lysozyme, DNase I, and complete EDTA-free protease inhibitor cocktail [Roche]). Cells were lysed on a French press using pressure at 15,000 lb/in^2^, and the cellular debris was removed by centrifugation. The supernatant was filtered through a 0.45-μm-pore-size syringe filter before being loaded onto a 1-ml HiTrap HF immobilized metal affinity chromatography (IMAC) column (GE Healthcare) using loading buffer, and the column was then washed with 10 ml of loading buffer (25 mM Tris [pH 7.5], 250 mM NaCl, 5 mM DTT, 30 mM imidazole). The recombinant His_6_-GFP-σ^A^ fusion protein was eluted from the column using a gradient of elution buffer (25 mM Tris [pH 7.5], 250 mM NaCl, 5 mM DTT, 500 mM imidazole). The fractions containing the fusion protein were pooled and dialyzed into TEV buffer (50 mM Tris [pH 7.5], 20 mM NaCl, 0.5 mM EDTA, 10% glycerol) using spin concentrators. The dialyzed protein was diluted to 1 mg ml^−1^ in the TEV buffer, and 1.5 mg of TEV protease was added. The reaction solution was incubated overnight at 4°C with agitation. The resulting σ^A^ and GFP proteins were separated using negative IMAC with the loading and elution buffers described above. The unbound fraction, containing σ^A^, was additionally purified using size exclusion chromatography with a Superdex75 resin (GE Healthcare) and buffer containing 25 mM Tris (pH 7.5) and 250 mM NaCl. The purified protein was concentrated to 1 mg ml^−1^ and sent for rabbit immunization to Dundee Cell Products (Dundee, United Kingdom). The obtained σ^A^ antiserum was affinity purified against purified recombinant σ^A^ according to a previously described protocol (41).

### Secondary-structure prediction.

Primary protein sequences of *S*. Typhimurium FlgN and B. subtilis FlgN were aligned using Clustal Omega (http://www.ebi.ac.uk/Tools/msa/clustalo/) (42). The secondary structures were predicted using PsiPred (http://bioinf.cs.ucl.ac.uk/psipred/) (43, 44) and aligned against the primary sequence of the proteins.

### RNA extraction and RT-PCR.

RNA isolation was carried out as described previously (38) using a RiboPure Bacteria RNA Isolation Kit (Ambion), according to the manufacturer's instructions, and treated with DNase I. To confirm cotranscription of *flgN* with *flgK* and *flgL*, cDNA was synthesized using the *yviE* gene-specific primer NSW1459 in a reaction with SuperScript III (Life Technologies) and subsequently treated with RNase H (NEB) for 20 min at 37°C. To establish if deletion of *flgN* perturbed transcription of *flgK* or *flgL* (and vice versa), cDNA was synthesized using random hexamers in a reaction with SuperScript III (Life Technologies). To amplify internal gene products, the following primer pairs were used: DEN5 and DEN7 (rRNA), NSW1446 and NSW1447 (*flgL*), NSW1444 and NSW1445 (*flgK*), NSW1442 and NSW1443 (*flgN*), and NSW1440 and NSW1441 (*flgM*).

### Motility assays.

Swimming and swarming analyses were performed as described previously (37) using low-salt LB medium (5 g liter^−1^ NaCl, 5 g liter^−1^ yeast extract, 10 g liter^−1^ tryptone) supplemented with 0.4% and 0.7% Bacto agar, respectively. Plates were incubated at 37°C, and the extent of swimming or swarming was noted at defined time intervals.

### Staining of flagella and fluorescence microscopy.

Cells carrying the Hag T^209^C point mutation (45) were grown to mid-exponential phase, and 0.5 ml of cells was harvested by centrifugation at 4,000 × *g*. Cells were washed once with 1× T-Base [1 mM EDTA, 15 mM (NH_4_)_2_SO_4_, 80 mM K_2_HPO_4_, 44 mM KH_2_PO_4_, 3.4 mM sodium citrate], pelleted, resuspended in 50 μl of T-Base containing 5 μg/ml Alexa Fluor 488 C_5_ maleimide dye (Molecular Probes), and incubated for 5 min at room temperature. Cells were washed three times with 500 μl of 1× T-Base and suspended in 50 μl of 1× phosphate-buffered saline (PBS). Two microliters of the cell suspension was spotted onto a thin matrix of 1.5% agarose in water (Ultrapure Agarose; Invitrogen) contained in a 1.7- by 2.8-cm Gene Frame (AB-0578; ABgene House, Epsom, Surrey, United Kingdom) mounted on a standard microscope slide (Super Premium slides; VWR). Each slide was prepared as follows: the gene frame was filled with molten 1.5% agarose and covered firmly with a standard microscope slide to flatten the agarose surface. Following solidification of the agarose, the slide was carefully removed, and the cell suspension was added. Once the cell suspension was dry, the gene frame was sealed with a coverslip (thickness number 1.5; VWR), and images were immediately acquired. Imaging was performed using a DeltaVision Core wide-field microscope (Applied Precision) mounted on an Olympus lX71 inverted stand with an Olympus 100× (1.4 numerical aperture [NA]) lens and a CoolSNAPHQ camera (Photometrics) with differential interference contrast (DIC) and fluorescence optics. GFP was imaged using a 100 W Mercury lamp and a fluorescein isothiocyanate (FITC) filter set (excitation, 490/20 nm; emission, 528/38 nm) with an exposure time of 200 ms. DIC images were illuminated with an LED-transmitted light source.

To monitor *P_hag_-yfp* expression, cells were grown at 37°C in LB medium to an OD_600_ of 1.0, 0.5 ml of the culture was harvested, and cells were washed and resuspended in 1× PBS. The cell suspension was prepared for microscopy and imaged as described above. Yellow fluorescent protein (YFP) fluorescence was imaged using a 100 W Mercury lamp and an FITC filter set (excitation, 490/20 nm; emission, 528/38 nm) with an exposure time of 50 ms. The threshold used to define activation of the transcriptional reporter *P_hag_* was set as a YFP fluorescence intensity value greater than 2 standard deviations above the mean background fluorescence. All images were rendered and analyzed postacquisition using OMERO software (www.openmicroscopy.org) (46).

### Flow cytometry analysis.

The fluorescence of strains harboring *yfp* or *gfp* transcriptional promoter fusions was measured in single cells extracted from planktonic cultures grown to mid-exponential phase and analyzed as described previously (47).

### Whole-cell analysis of Hag.

Proteins were extracted from planktonic cultures grown to mid-exponential phase. Briefly, cells were harvested by centrifugation at 4,700 × *g*. Cells were suspended in 1× Bugbuster (Novagen) and lysed according to the manufacturer's instructions. Seven micrograms of protein was resolved by SDS-PAGE and stained with Coomassie brilliant blue. Hag was identified by comparison with the Δ*hag* strain (DS1677) and confirmed by mass spectrometry (FingerPrints Proteomics and Mass Spectrometry Facility, University of Dundee).

### Western blot analysis.

Cellular proteins were extracted as for whole-cell analysis of Hag. Extracellular proteins were extracted from the culture supernatant and processed as detailed previously (48) and suspended in 50 μl of 4× SDS loading dye. Seven micrograms of cellular proteins or 7 μl of extracellular proteins was separated by SDS-PAGE prior to transfer onto polyvinylidene difluoride (PVDF) membrane (Millipore) by electroblotting. Antibodies raised against Hag (a kind gift from Kürsad Turgay) were used at 1:40,000, anti-FlgE (a kind gift from Daniel Kearns) was used at 1:20,000, anti-σ^A^ was used at 1:500, and goat anti-rabbit or goat anti-mouse horseradish peroxidase (HRP)-conjugated secondary antibodies (both from Pierce) were used at 1:5,000.

### β-Galactosidase assays.

The β-galactosidase activity of strains harboring *lacZ* promoter reporter fusions was measured as previously described (37, 49). The values presented are the average β-galactosidase activities in Miller units (50) determined from at least three independent samples. Error bars represent the standard errors of the means.

### Enrichment of flagellar hook-basal bodies.

The flagellar HBB fraction of NCIB3610 was enriched for as described previously (51, 52). Briefly, 1 liter of cells was grown to early exponential phase and harvested by centrifugation at 6,000 × *g* for 45 min at 4°C. The cell pellet was resuspended in 100 ml of sucrose solution (0.5 M sucrose, 0.15 M Tris) with a protease inhibitor tablet (Roche), and cells were homogenized with a loose pestle on ice. To lyse cells and allow spheroplast formation, lysozyme was added to the cell suspension at a final concentration of 0.1 mg ml^−1^, and samples were incubated at 4°C with stirring for 40 min. Spheroplasts were lysed by addition of Triton X-100 to a final concentration of 1%, and viscosity was decreased by stirring at room temperature for 30 min, allowing endogenous DNases to degrade cellular DNA. Unlysed cells were removed by centrifugation at 4,000 × *g* for 10 min, and EDTA was added to the suspension at a final concentration of 10 mM. To aid removal of contaminating membrane proteins, the pH of the lysate was raised to 10 by addition of NaOH. The lysate then underwent high-speed centrifugation (60,000 × *g* for 60 min), and the pellets were resuspended in alkaline solution (0.1 M KCl-KOH, 0.5 M sucrose, 0.1% Triton X-100, pH 11.0) and centrifuged again. The pellet was then resuspended in 90 ml of TET buffer (10 mM Tris-HCl, 5 mM EDTA, 0.1% Triton X-100, pH 8.0), and a 36% CsCl gradient was established. The solution was centrifuged using a Beckman SW41Ti swinging-bucket rotor at 55,000 × *g* for 16 h at 15°C. The flagellar fraction was visible as a band approximately 2 cm from the bottom of the tube and was collected with a Pasteur pipette and dialyzed against TET buffer. In an attempt to dissociate the flagellar filaments, the flagellar fraction was suspended in acidic solution (glycine-HCl, 0.1% Triton X-100, pH 3.0) for 1 h, and HBB complexes were collected by centrifugation at 100,000 × *g* for 1 h. HBBs were resuspended in TET buffer and analyzed by mass spectrometry. Results were searched against the Bacillus subtilis Mascot database (FingerPrints Proteomics and Mass Spectrometry Facility, University of Dundee). The top 12 proteins as identified by their Mascot scores are listed in Table S4 in the supplemental material.

## RESULTS

### YvyG of B. subtilis shares secondary-structure homology with the Salmonella protein FlgN.

**FIG 1:**
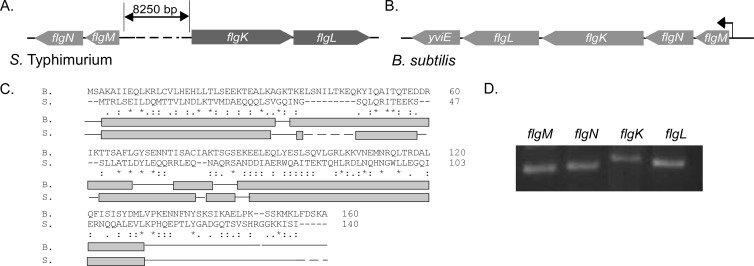
Primary-sequence and secondary-structure comparison of B. subtilis FlgN with *S*. Typhimurium FlgN. Schematic representations of the chromosomal regions surrounding *S*. Typhimurium *flgN* (A) and B. subtilis *flgN* (B) are shown. Arrows represent open reading frames, with the direction of the arrow indicating the direction of the open reading frame. The bent arrow represents the promoter located before the *flgM* coding region. (C) Primary-sequence alignment and secondary-structure comparison of *Bs-flgN* and *S*T-*flgN*. The primary amino acid sequence of FlgN from B. subtilis 168 (B) was aligned with that of *flgN* from *S*. Typhimurium (S). For primary-sequence alignments, an asterisk indicates a fully conserved amino acid, a colon indicates a highly conserved amino acid, and a period indicates a weakly conserved amino acid. Gaps indicate no homology. Dashed lines indicate a break in the sequence. For secondary-structure alignments light gray boxes indicate α-helices, solid lines indicate coiled coils, and dashed lines indicate a break in the sequence alignment. (D) RT-PCR analysis of cotranscription of *flgM*, *flgN*, *flgL*, and *flgK*. Regions of DNA internal to *flgM*, *flgN*, *flgK*, and *flgL* were amplified from cDNA generated using a primer specific to the gene 3′ proximal to *flgL*, using RNA from the wild-type strain (NCIB3610).

Previous bioinformatic analysis postulated that YvyG of B. subtilis was an orthologue of FlgN (27). FlgN is an essential component of the flagellar type III secretion machinery (26, 36, 53). FlgN has been most extensively studied in *S*. Typhimurium (26, 36), but homologues have been recognized in a number of different bacterial species (27, 53). Three criteria were employed by Pallen et al. (27) to identify FlgN homologues in a broad range of bacterial species: (i) the protein must be encoded by a gene adjacent to an *flgM* homologue (Fig. 1A and B), (ii) the gene should be of a similar length to *flgN* of Salmonella (Fig. 1C), and (iii) the protein must be recognizable (however distantly) by PSI-BLAST analysis as “FlgN-like.” These criteria were set as the primary amino acid sequence of FlgN is highly variable (27). To confirm that YvyG is likely to be an orthologue of FlgN, the amino acid sequence of YvyG was compared with that of *S*. Typhimurium FlgN. As expected, there was little primary-sequence homology (Fig. 1C). However, upon comparison of the predicted secondary structures of YvyG and FlgN, a high degree of similarity was clearly apparent (Fig. 1C). Thus, YvyG was renamed FlgN, and its function in motility in B. subtilis was further investigated.

On the B. subtilis chromosome, the *flgM*, *flgN*, *flgK*, and *flgL* coding regions are adjacent. To ascertain whether all four genes were part of the same operon in the NCIB3610 strain, reverse transcription-PCR analysis was used. RNA was extracted from wild-type B. subtilis, and cDNA was synthesized using a primer specific to *yviE*, the gene proximal to *flgL* at the 3′ end (Fig. 1B). The cDNA generated was used as a template for PCR with primer pairs specific to the internal coding regions of *flgM*, *flgN*, *flgK*, and *flgL*. Regions of DNA internal to the coding regions of *flgM*, *flgN*, *flgK*, and *flgL* could each be amplified from cDNA generated using a primer at the 3′ end of *flgL* (just beyond the termination codon) (Fig. 1D), demonstrating that all four genes are cotranscribed.

### FlgN is essential for swarming and swimming motility of B. subtilis.

**FIG 2:**
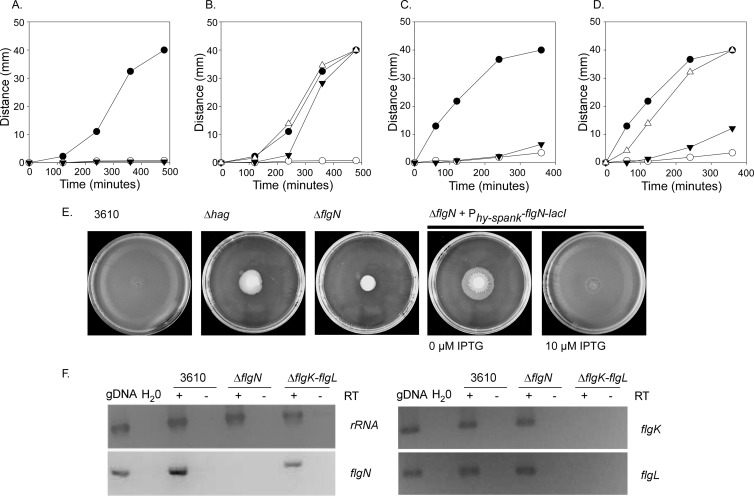
Δ*flgN* strains are nonmotile. Swim expansion assays (A and B) and swarm expansion assays (C and D) were performed. Wild-type (3610; filled circle), Δ*hag* (DS1677; filled triangle), and Δ*flgN* (NRS3570; open circle) strains were used for the experiments shown in panels A and C. Wild-type (3610; filled circle) and Δ*flgN* (NRS3570; open circle) strains along with the Δ*flgN amyE*::P_*hy-spank*_-*flgN-lacI* (NRS3578) strain without (filled triangle) and with (open triangle) 10 μM IPTG induction were used for the experiments shown in panels B and D. Each graph is representative of three independent biological replicates. (E) Photographs of swarm expansion plates taken at the end of the assay, after 6 h of incubation at 37°C. (F) RT-PCR analysis of transcription of rRNA, *flgN*, *flgK*, and *flgL* in wild-type (NCIB3610), Δ*flgN* (NRS3570), and Δ*flgK-flgL* (NRS4060) strains. Genomic DNA (gDNA) and H_2_O are shown as positive and negative controls for amplification, respectively. Reaction mixtures were incubated with (+) or without (−) reverse transcriptase (RT).

The *flgN* gene is located in a region of the chromosome known to be required for motility (9). To test if *flgN* is required for motility, a B. subtilis strain carrying an in-frame deletion of *flgN* was constructed, and its phenotype was assessed in swimming and swarming assays. A strain containing a deletion in the *hag* gene (DS1677) was used as a nonmotile control. While the wild-type strain was able both to swim and swarm efficiently (Fig. 2A, C, and E), this behavior was lost in the Δ*flgN* strain (NRS3570) (Fig. 2A, C, and E). Using reverse transcription-PCR analysis, we confirmed that transcription of the other genes in the operon was not impacted by the *flgN* deletion (Fig. 2F). To further ensure that this loss of motility was specific to the deletion of *flgN*, the motility of a strain where the coding region of *flgN* was reintroduced at the *amyE* locus under the control of an IPTG-inducible promoter (P_*hy-spank*_) was tested. Both the swimming (Fig. 2B) and swarming (Fig. 2D and E) phenotypes of the Δ*flgN* strain could be restored by the reintroduction of *flgN* on the chromosome upon induction with IPTG. These data demonstrate for the first time *in vivo* that the protein product of the *flgN* gene is required for both swimming and swarming motility in B. subtilis. It is notable that higher levels of *flgN* transcription are required to complement the swarming defect presented by the Δ*flgN* strain than are needed for the swimming defect (compare Fig. 2B and D). This is most likely attributable to a higher demand for flagellum biosynthesis in swarming than swimming motility (54).

### Deletion of *flgN* results in a loss of bimodal transcription of *hag*.

**FIG 3:**
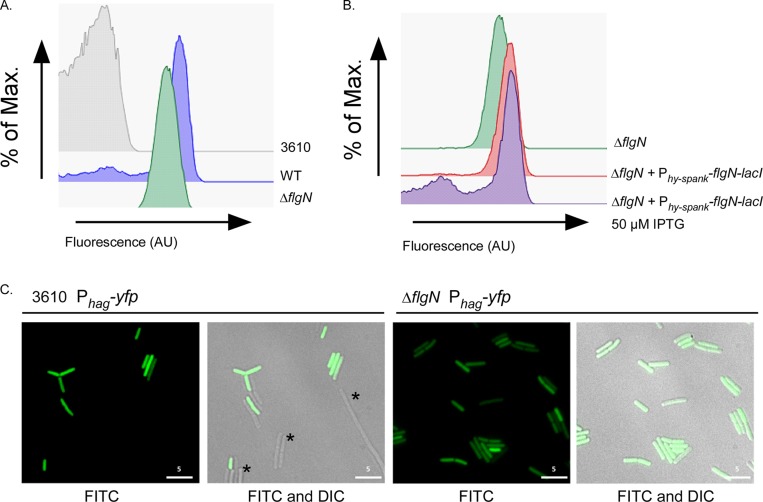
Deletion of *flgN* results in a loss of bimodal *hag* transcription. (A and B) Flow cytometry analysis of *hag* transcription in strains carrying the P_*hag*_-*yfp* transcriptional reporter fusion. Strain 3610 was used as a nonfluorescent control. Shown are the wild-type (WT; NRS3076), Δ*flgN* (NRS3570), and Δ*flgN amyE*::P_*hy-spank*_-*flgN-lacI* (NRS3713) strains without and with induction with 50 μM IPTG. (C) Fluorescence microscopy analysis of the wild-type (NRS3076) and Δ*flgN* (NRS3708) strains carrying the P_*hag*_-*yfp* transcriptional reporter (false-colored green). Scale bar, 5 μm. Asterisks indicate examples of cells that do not transcribe *hag*. Max, maximum; AU, arbitrary units.

To dissect the role of *flgN* in motility, the effect of deletion of *flgN* on the transcription of the flagellar filament gene *hag* was tested. Due to the heterogeneity in *sigD* transcription, transcription of *hag* is bimodal, and thus single-cell techniques are ideally suited for analysis (11, 12). To this end, a P_*hag*_-*yfp* transcriptional reporter was integrated at a heterologous location on the chromosome. Flow cytometry and single-cell microscopy were used to assess the transcription profile using the fluorescence generated by YFP as a reporter. In the wild-type strain, the bimodality of *hag* transcription in the cell population is clearly evident (Fig. 3A and C). Strikingly, upon deletion of *flgN* bimodality is lost as *hag* transcription was observed for all cells within the population, albeit at a slightly lower level than in the wild type (Fig. 3A and C). The alteration in the *hag* transcription profile can be complemented upon reintroduction of *flgN* under the control of an IPTG-inducible promoter at the heterologous *amyE* locus, confirming the requirement of *flgN* for bimodal *hag* transcription (Fig. 3B). However, as the *hag* gene is still transcribed in the absence of *flgN*, these findings indicate that lack of motility in the Δ*flgN* strain is not due to a lack of *hag* transcription.

### Loss of *flgN* is associated with a defect in flagellar biosynthesis.

**FIG 4:**
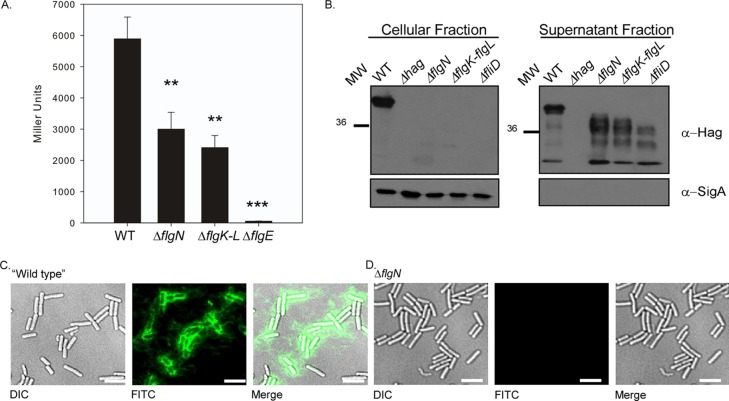
Deletion of *flgN* is associated with a decrease in *hag* translation and a block in filament assembly. (A) β-Galactosidase assays of strains carrying the P*hag*′-′*lacZ* translational reporter fusion. Shown are the wild-type (WT; NRS4795), Δ*flgN* (NRS4796), Δ*flgK-flgL* (NRS4799), and Δ*flgE* (NRS4798) strains. Data are plotted as the averages of at least three independent replicates. Error bars represent standard errors of the means. Asterisks denote significance as calculated by a Student *t* test: *, *P* < 0.05; **, *P* < 0.01; ***, *P* < 0.001. (B) Western blot analysis of cellular (including assembled flagella) and supernatant (including sheared and unassembled flagella) fractions of the wild-type (3610), Δ*hag* (DS1677), Δ*flgN* (NRS3570), Δ*flgK-flgL* (NRS4060), and Δ*fliD* (NRS4041) strains, separately probed with anti-Hag and anti-σ^A^ primary antibodies. MW, molecular weight in thousands; α, anti. (C and D) Fluorescence micrographs of strains carrying the Hag T^209^C point mutation labeled with Alexa Fluor 488 C_5_ maleimide (false-colored green). Shown are the wild-type (NRS3719) (C) and Δ*flgN* (NRS3718) (D) strains. Scale bar, 5 μm.

Given that the *hag* coding region is transcribed (Fig. 3), a lack of motility in the absence of *flgN* could arise from lack of translation of the *hag* transcript. To assess *hag* translation, a P*hag*′-′*lacZ* translational reporter fusion (under the control of the *hag* promoter, the *hag* leader region, Shine-Dalgarno sequence, and start codon) was constructed and introduced into B. subtilis at the *amyE* locus. When the β-galactosidase activity levels in the *flgN* mutant were compared with the wild-type levels, Hag translation was found to be 2-fold lower (Fig. 4A). However, a strain carrying an in-frame deletion of *flgE*, which encodes the main protein component of the flagellar hook, showed a 100-fold decrease in Hag translation (Fig. 4A) (55). It is likely that the observed decrease in the translation of *hag* in the Δ*flgN* mutant strain is a result of *hag* being transcribed at a slightly lower level in all cells than in the wild-type strain (Fig. 3). Alternatively, it is technically possible that translation itself may be regulated, as occurs in the absence of *flgE* (see Discussion) (55). While there is a statistically significant (*P* = 0.01) decrease in Hag translation in the absence of *flgN*, it did not appear to be sufficient to account for the complete lack of motility demonstrated in Fig. 2 (i.e., the severity of the motility defect does not match the small decrease in translation of Hag). A lack of motility in the presence of *hag* transcription and translation could be due to a lack of Hag polymerization. To test if Hag was secreted but not assembled into a flagellar filament in the Δ*flgN* strain, proteins were extracted from the cellular and supernatant fractions of cells grown to mid-exponential phase. The presence or absence of Hag was detected by Western blotting, with the cytoplasmic sigma factor, σ^A^, used as a loading and fractionation control. For the wild-type strain Hag is detected in the cellular fraction (which includes assembled flagella) and in the supernatant fraction (including unassembled and sheared flagella) (Fig. 4B). However, for the Δ*flgN* strain Hag is present only in the supernatant fraction at a lower molecular weight (Fig. 4B). This is likely to be unpolymerized Hag or the products of proteolytic degradation resulting from the action of the extracellular proteases (13). As a positive control for Hag secretion, a strain carrying an in-frame deletion of *fliD*, which encodes the filament cap protein, was also assessed. As shown previously (13), this strain phenocopies the Hag secretion profile seen in the Δ*flgN* strain, thus supporting our conclusions.

Loss of filament polymerization in the Δ*flgN* strain was confirmed by single-cell fluorescence microscopy of strains where the codon for threonine at position 209 of the *hag* gene was mutated to cysteine to enable labeling with an Alexa Fluor 488 C_5_ maleimide dye (45). In the wild-type background (NRS3719) the flagellar filaments are clearly visible (Fig. 4C). However, upon deletion of *flgN*, no signal was detected (Fig. 4D). Collectively, these data show that the Δ*flgN* strain is nonmotile due to both a small decrease in translation of Hag and a block in filament assembly, which results in the accumulation of unpolymerized Hag in the extracellular milieu. Together, these data prove that the lack of motility exhibited by the *flgN* mutant is due to a lack of flagellar filament assembly.

### Deletion of the hook junction genes generates a strain which phenocopies the *flgN* mutant.

**FIG 5:**
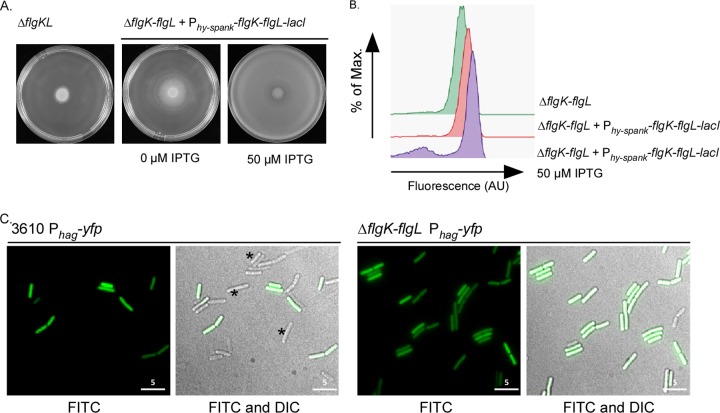
The Δ*flgN* strain phenocopies the Δ*flgK-flgL* strain. (A) Photographs of swarm expansion plates taken after 6 h of incubation at 37°C. Shown are the wild-type (3610), Δ*flgK-flgL* (NRS4060), and Δ*flgK-flgL amyE*::P_*hy-spank*_-*flgK-flgL-lacI* (NRS 4064) strains without and with induction with 50 μM IPTG. (B) Flow cytometry analysis of *hag* transcription in strains carrying the P_*hag*_-*yfp* transcriptional reporter fusion. Shown are the Δ*flgK-flgL* (NRS4071) and Δ*flgK-flgL amyE*::P_*hy-spank*_-*flgK-flgL-lacI* (NRS4078) strains without and with induction with 50 μM IPTG. (C) Fluorescence microscopy analysis of the wild-type (NRS3076) and Δ*flgK-flgL* (NRS4071) strains carrying the P_*hag*_-*yfp* transcriptional reporter (false-colored green). Scale bar, 5 μm. Asterisks indicate examples of cells that do not transcribe *hag*.

A lack of filament polymerization in the absence of *flgN* is consistent with the hypothesis that *Bs*-FlgN is an orthologue of *S*T-FlgN; i.e., if FlgK and FlgL are not properly localized to the hook-filament junction, the flagellar filament cannot be assembled (26, 53). We proposed that if *Bs*-FlgN were indeed an orthologue of *S*T-FlgN, then a B. subtilis strain lacking *flgK* and *flgL* might phenocopy the Δ*flgN* strain. A strain carrying an in-frame deletion of *flgK-flgL* was constructed and found to be unable to swarm (Fig. 5A). In addition, flow cytometry and microscopy analyses revealed that, like deletion of *flgN*, deletion of *flgK-flgL* resulted in a loss of bimodality with respect to transcription of the σ^D^-regulated gene *hag* (Fig. 5B and C) and a 2-fold decrease in Hag translation compared with the wild-type strain (Fig. 4A). Furthermore, upon deletion of *flgK-flgL*, Hag could not be detected in the cellular fraction but was instead found in the extracellular milieu (Fig. 4B). Using reverse transcription-PCR analysis, we confirmed that transcription of the other genes in the operon was not impacted by the *flgK-flgL* deletion (Fig. 2F). All of the phenotypes were proven to be specific to deletion of the *flgK-flgL* coding region as the mutant strain could be complemented by replacement of the *flgK-flgL* coding region at a heterologous location on the chromosome under the control of the P_*hy-spank*_ promoter (Fig. 5A and B). In conclusion, a double deletion of *flgK* and *flgL* generates a strain that phenocopies the *flgN* mutant, as demonstrated by physiological, biochemical, and single-cell analyses. This is consistent with *Bs*-FlgN functioning as an orthologue of *S*T-FlgN.

### Overexpression of *flgK-flgL* cannot compensate for the absence of *flgN*.

**FIG 6:**
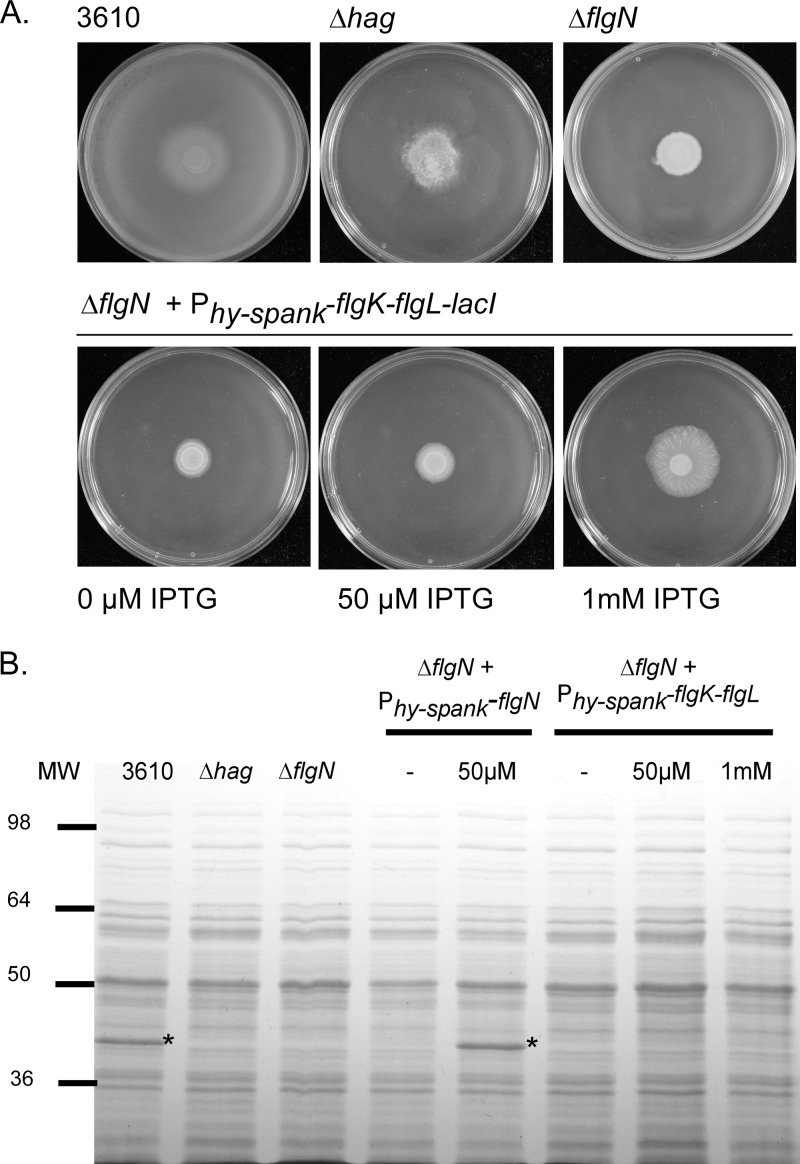
Overexpression of *flgK-flgL* cannot complement a Δ*flgN* mutant. (A) Photographs of swarm expansion plates taken after 6 h of incubation at 37°C. Shown are the wild-type (3610), Δ*hag* (DS1677), Δ*flgN* (NRS3570), and Δ*flgN amyE*::P_*hy-spank*_-*flgK-flgL-lacI* (NRS4043) strains without and with induction with 50 μM IPTG or 1 mM IPTG. (B) Coomassie gel analysis of cellular fractions of the 3610, Δ*hag* (DS1677), Δ*flgN* (NRS3570), Δ*flgN amyE*::P_*hy-spank*_-*flgN-lacI* (NRS3578), and Δ*flgN amyE*::P_*hy-spank*_-*flgK-flgL-lacI* (NRS4043) strains without and with induction with 50 μM IPTG or 1 mM IPTG. The Hag protein was subsequently identified by mass spectrometry analysis and is marked with asterisks. MW, molecular weight in thousands.

In Salmonella deletion of *flgN* can be compensated for by overexpression of *flgK* and *flgL* (16). This is because FlgN is not exclusively required for the secretion of its substrates but rather protects FlgK and FlgL from proteolysis and ensures that the substrates are efficiently transported to the export machinery (16, 17). To test if this was the case for the B. subtilis *flgN* deletion, the coding regions of *flgK* and *flgL* were integrated in the Δ*flgN* strain at a heterologous site on the chromosome under the control of an IPTG-inducible promoter. Induction of the *flgK-flgL* coding region was unable to restore motility to the Δ*flgN* strain, as determined by assaying swarming motility (Fig. 6A). To confirm that the flagellar filament was not polymerized upon overexpression of *flgK-flgL*, cellular protein samples (which include assembled flagella) were separated by SDS-PAGE and stained with Coomassie brilliant blue. Hag appears as a dominant protein band at ∼36 kDa (Fig. 6B) and can be easily identified by comparison with proteins harvested from the Δ*hag* and wild-type strains (56). Moreover, the identity of the Hag protein was confirmed by mass spectrometry (see Fig. S1 in the supplemental material). Compared with the wild type, analysis of the cellular proteins for the Δ*flgN* strain indicated that Hag was not associated with the cell fraction (Fig. 6B). This is entirely consistent with the data presented above (Fig. 4 and 6A). As expected, the presence of the Hag band could be restored by the reintroduction of *flgN* on the chromosome upon induction with 50 μM IPTG (Fig. 6B). However, introduction of *flgK-flgL* in the Δ*flgN* background at a heterologous site could not complement the Δ*flgN* mutant with respect to Hag polymerization, even in the presence of 1 mM IPTG. The inability of *flgK-flgL* overexpression to compensate for deletion of *flgN* could be due to disruption of flagellar biosynthesis at an earlier stage. However, this possibility was ruled out as we demonstrated by Western blotting that the flagellar hook protein, FlgE, was detected in whole-cell lysates (which include assembled flagella) for both the wild-type and Δ*flgN* strains (see Fig. S2 in the supplemental material).

The inability of heterologous *flgK-flgL* expression to complement the *flgN* mutant strain is suggestive of a strict dependence on FlgN for FlgK-FlgL protein stability or secretion in B. subtilis. In an attempt to test if FlgK was unstable in the absence of *flgN*, strains were constructed to enable detection of FlgK by fusing FlgK to a poly histidine epitope tag. However, the presence of such an epitope tag at either the N or C terminus of the protein rendered FlgK nonfunctional, as determined by a nonmotile phenotype (see Fig. S3A in the supplemental material). As a method of attempting to assess FlgK assembly at the flagellar hook junction, we aimed to purify flagellar hook-basal bodies from both the wild-type and Δ*flgN* strains and to analyze the protein components of the complex by mass spectrometry. While we were successfully able to enrich the flagellar fraction of the wild-type strain (see Fig. S3B and Table S4 in the supplemental material), we were unable to do so for the Δ*flgN* strain. This is most likely because this methodology is dependent on an intact flagellar filament for isolation of the complex, and the *flgN* mutant does not form a flagellar filament (Fig. 4). Finally, in *S*. Typhimurium, FlgN interacts directly with FlgK and FlgL to protect the proteins from proteolytic cleavage (25). To test if FlgN could interact with either FlgK or FlgL to perform a similar role in B. subtilis, bacterial two-hybrid experiments were undertaken. However, an interaction could not be detected (data not shown), which could be due to inactivity of the fusion protein. Thus, despite extensive efforts, we were unable to determine the stability of FlgK in the absence of *flgN* or if there was an interaction between the proteins.

### *In vivo* analysis of the role of phosphorylation in controlling motility.

**FIG 7:**
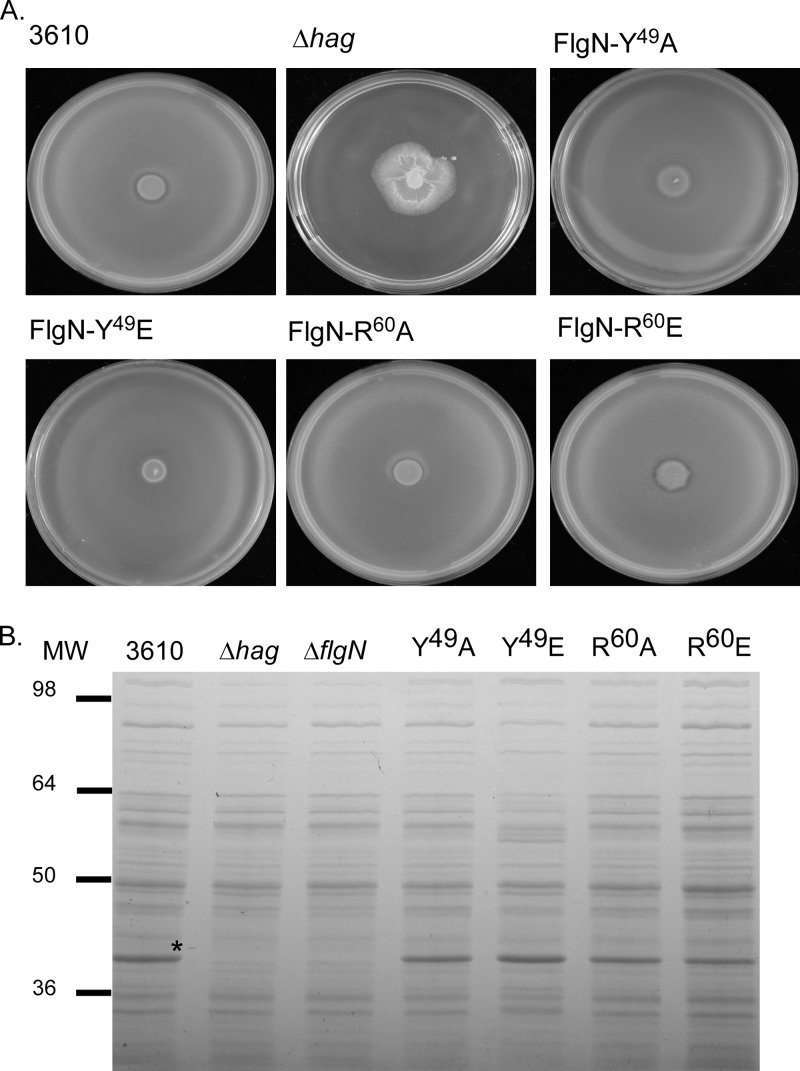
Mutation of identified FlgN phosphorylation sites does not affect motility. (A) Photographs of swarm expansion plates taken after 6 h of incubation at 37°C. Shown are the wild-type (3610), Δ*hag* (DS1677), *flgN*-Y^49^A (NRS3571), *flgN*-Y^49^E (NRS3724), *flgN*-R^60^A (NRS4063), and *flgN*-R^60^E (NRS4017) strains. (B) Coomassie gel analysis of cellular fractions of the 3610, Δ*hag* (DS1677), *flgN*-Y^49^A (NRS3571), *flgN*-Y^49^E (NRS3724), *flgN*-R^60^A (NRS4063), and *flgN*-R^60^E (NRS4017) strains. The Hag protein is marked with asterisks. MW, molecular weight in thousands.

Global proteomic strategies have identified a plethora of targets for both tyrosine and arginine kinases in B. subtilis (28–30). One such target is FlgN, which can be tyrosine phosphorylated on amino acid 49 (29) and arginine phosphorylated on amino acid 60 (30). Having demonstrated that FlgN is required for flagellar biosynthesis, we were presented with the ideal system to assess the role of tyrosine and arginine phosphorylation in controlling protein function *in vivo*. Site-directed mutagenesis was used to mutate the chromosomal copy of *flgN* tyrosine 49 to alanine (Y^49^A) to assess the effects of preventing phosphorylation. As shown in Fig. 7, swarming motility was not affected. We next tested the impact of replacing tyrosine 49 with glutamic acid (Y^49^E) to mimic the negative charge associated with phosphorylation. Once more, the strain containing the mutant allele of FlgN (Y^49^E) exhibited a motility phenotype that was indistinguishable from that of the wild type (Fig. 7A). Subsequently, strains were constructed where the coding region of *flgN* on the chromosome was mutated such that the arginine at position 60 was replaced with either alanine or glutamic acid. The motility phenotypes of these strains were tested, and they were found to swarm at the same rate as the wild-type strain (Fig. 7A). SDS-PAGE analysis of whole-cell lysates also showed that Hag was synthesized and that the cellular localization was directly comparable to that of the wild type for each of the point mutation strains (Fig. 7B). These data demonstrate that mutation of the previously defined tyrosine or arginine phosphorylation site has no discernible impact on the function of FlgN in B. subtilis NCIB3610.

## DISCUSSION

In this work we report that *yvyG* is required for the motility of B. subtilis. We demonstrate that the main role for YvyG is to enable flagellar filament polymerization. The data presented allow us to conclude that YvyG is indeed an orthologue of FlgN from *S*. Typhimurium, but in B. subtilis it would appear that there is a strict reliance on YvyG for the secretion and placement of FlgK and FlgL at the hook-filament junction. In light of these data, we suggest that YvyG be referred to as FlgN.

### The role of FlgN in the regulation of flagellum biosynthesis.

This work suggests that in B. subtilis FlgN partially mediates flagellum biosynthesis through its ability to regulate *hag* transcription and translation. In wild-type B. subtilis the sigma factor σ^D^ (*sigD*) needed for *hag* transcription is transcribed only in a subpopulation of cells, resulting in bimodal expression of *hag* (11, 12). Deletion of *flgN* results is *hag* being transcribed in every cell, albeit at a lower level (Fig. 3), indicating that σ^D^ is active in every cell in this genetic background. Consistent with this, we did not observe any cell chaining in the absence of *flgN*, indicating that the σ^D^-dependent autolysins (10) are also transcribed in all cells. It is known that σ^D^ can be regulated by transcription of the *sigD* gene and by interaction with the anti-sigma factor, FlgM (11). Therefore, the change in *hag* transcription observed upon deletion of *flgN* could be explained by a change in regulation by the anti-sigma factor, FlgM. In *S*. Typhimurium FlgM regulates the transcription of late-class σ^28^-regulated flagellar genes by both sequestering free σ^28^ and destabilizing the σ^28^ RNA polymerase holoenzyme complex (57). Upon completion of HBB assembly, FlgM is secreted, and σ^28^ is able to activate target promoters (58). It has been previously reported that FlgN is able to regulate the translation of FlgM in *S*. Typhimurium (59), therefore raising the possibility that in the B. subtilis Δ*flgN* strain translation of FlgM is decreased, allowing σ^D^ to trigger transcription of *hag* in all cells. The regulation of FlgM in B. subtilis is poorly understood, and so whether this is the case has yet to be determined.

Deletion of *flgN* in B. subtilis also results in a 2-fold decrease in *hag* translation (Fig. 4A). This effect could technically be due to translational regulation, as is seen for the Δ*flgE* strain (Fig. 4A) (55). Indeed, recent studies in B. subtilis have identified the RNA binding protein CsrA, the CsrA regulatory protein FliW, and the molecular chaperone FliS as having roles in controlling Hag translation or secretion (13, 55, 60). When cellular levels of Hag are depleted, FliW binds to CsrA, leaving it unable to occlude the *hag* Shine-Dalgarno sequence, allowing translation to proceed. However, when Hag protein accumulates in the cytoplasm, it is able to interact with and sequester FliW, resulting in CsrA-mediated repression of translation (55). Therefore, inhibition of translation by CsrA relies on accumulation of Hag within the cell. However, the data presented in Fig. 4B show that in the absence of *flgN*, Hag accumulates not in the cytoplasm but in the extracellular milieu. This not only suggests that CsrA is not responsible for the observed decrease in Hag translation but also is in keeping with the hypothesis that FlgN is required for the assembly of FlgK and FlgL; in the absence of the hook-filament junction, flagellin cannot be properly assembled and so accumulates in the extracellular milieu (53). For this reason we favor the hypothesis that the change in the transcriptional profile of *hag* is responsible for the decrease in translation observed.

### The role of FlgN phosphorylation.

Tyrosine and arginine phosphorylation events have been implicated in the control of diverse biological processes in B. subtilis, including biofilm formation (38, 61), DNA replication (62), exopolysaccharide synthesis (63), the heat shock response (64), and potentially the regulation and/or assembly of the flagellar filament (30). The motility protein FlgN has been shown to be both tyrosine and arginine phosphorylated (29–31). Moreover, the subcellular localization of FlgN was reported to be impacted by deletion of the tyrosine kinase PtkA (31). However, site-directed mutagenesis of the reported FlgN tyrosine and arginine phosphorylation sites *in vivo* failed to impact the motility of B. subtilis (Fig. 7). These findings led us to conclude that a dominant role for phosphorylation of these residues does not exist. When these findings are considered in a wider context of the function of posttranslational modifications, they may not be surprising. For instance, in eukaryotes, it has been suggested that many phosphorylation events are nonfunctional or may occur at a very low stoichiometry such that they do not impact the function of the protein (65, 66). Indeed, two tyrosine kinases (61, 63) and one arginine kinase (30) have been identified in B. subtilis, but many more proteins have been identified as being phosphorylated, thereby suggesting that each kinase is promiscuous. This may imply that random encounters between kinases and phosphorylatable sites on different proteins might result in nonspecific and nonfunctional phosphorylation events. Alternatively, FlgN might act as a phosphate sink or store to remove free phosphate from the system (67).

### Concluding remarks.

The regulation and biosynthesis of the bacterial flagellum are best understood for Gram-negative bacterial species such as *S*. Typhimurium. However, recent work on B. subtilis has begun to illuminate how Gram-positive bacterial species are able to coordinate flagellar assembly. These studies have uncovered key differences between Gram-negative and Gram-positive bacterial flagella as well as having highlighted many conserved mechanisms. The data presented here illustrate that FlgN from B. subtilis is essential for flagellar filament polymerization and therefore motility. We propose that *Bs*-FlgN is an orthologue of the *S*. Typhimurium chaperone protein FlgN and is required for the export and assembly of FlgK and FlgL at the hook-filament junction. By way of contrast, in a B. subtilis strain lacking *flgN* flagella are not detectable, and overexpression of *flgK-flgL* is not sufficient to overcome the motility defect exhibited by the Δ*flgN* strain. Therefore, while *Bs*-FlgN bears great functional similarity to *S*T-FlgN, there are crucial differences which suggest that there is a stricter dependence on FlgN for the export of FlgK and FlgL in B. subtilis. Overall, this work further emphasizes the previously underappreciated differences in flagellar gene regulation between Gram-positive and Gram-negative bacterial species.

## Supplementary Material

Supplemental material

## References

[1] Chevance FF Hughes KT 2008 Coordinating assembly of a bacterial macromolecular machine. Nat. Rev. Microbiol. 6:455–465. 10.1038/nrmicro1887 18483484 PMC5963726

[2] O'Toole GA Kaplan HB Kolter R 2000 Biofilm formation as microbial development. Annu. Rev. Microbiol. 54:49–79. 10.1146/annurev.micro.54.1.49 11018124

[3] Cairns LS Marlow VL Bissett E Ostrowski A Stanley-Wall NR 2013 A mechanical signal transmitted by the flagellum controls signalling in Bacillus subtilis. Mol. Microbiol. 90:6–21. 10.1111/mmi.12342 23888912 PMC3963450

[4] Serra DO Richter AM Klauck G Mika F Hengge R 2013 Microanatomy at cellular resolution and spatial order of physiological differentiation in a bacterial biofilm. mBio. 4:e00103–13. 10.1128/mBio.00103-13 23512962 PMC3604763

[5] Marquez-Magana LM Chamberlin MJ 1994 Characterization of the *sigD* transcription unit of Bacillus subtilis. J. Bacteriol. 176:2427–2434 8157612 10.1128/jb.176.8.2427-2434.1994 PMC205368

[6] West JT Estacio W Marquez-Magana L 2000 Relative roles of the *fla/che* P_A_, P_D-3_, and P_*sigD*_ promoters in regulating motility and *sigD* expression in Bacillus subtilis. J. Bacteriol. 182:4841–4848. 10.1128/JB.182.17.4841-4848.2000 10940026 PMC111362

[7] Helmann JD Marquez LM Chamberlin MJ 1988 Cloning, sequencing, and disruption of the Bacillus subtilis sigma 28 gene. J. Bacteriol. 170:1568–1574 2832368 10.1128/jb.170.4.1568-1574.1988 PMC211003

[8] Chen YF Helmann JD 1992 Restoration of motility to an Escherichia coli *fliA* flagellar mutant by a Bacillus subtilis sigma factor. Proc. Natl. Acad. Sci. U. S. A. 89:5123–5127. 10.1073/pnas.89.11.5123 1594620 PMC49241

[9] Mirel DB Lauer P Chamberlin MJ 1994 Identification of flagellar synthesis regulatory and structural genes in a sigma D-dependent operon of Bacillus subtilis. J. Bacteriol. 176:4492–4500 8045879 10.1128/jb.176.15.4492-4500.1994 PMC196267

[10] Marquez LM Helmann JD Ferrari E Parker HM Ordal GW Chamberlin MJ 1990 Studies of sigma D-dependent functions in Bacillus subtilis. J. Bacteriol. 172:3435–3443 2111808 10.1128/jb.172.6.3435-3443.1990 PMC209155

[11] Cozy LM Kearns DB 2010 Gene position in a long operon governs motility development in Bacillus subtilis. Mol. Microbiol. 76:273–285. 10.1111/j.1365-2958.2010.07112.x 20233303 PMC2911795

[12] Kearns DB Losick R 2005 Cell population heterogeneity during growth of Bacillus subtilis. Genes Dev. 19:3083–3094. 10.1101/gad.1373905 16357223 PMC1315410

[13] Mukherjee S Babitzke P Kearns DB 2013 FliW and FliS function independently to control cytoplasmic flagellin levels in Bacillus subtilis. J. Bacteriol. 195:297–306. 10.1128/JB.01654-12 23144244 PMC3553831

[14] Titz B Rajagopala SV Ester C Hauser R Uetz P 2006 Novel conserved assembly factor of the bacterial flagellum. J. Bacteriol. 188:7700–7706. 10.1128/JB.00820-06 16936039 PMC1636259

[15] Bennett JC Hughes C 2000 From flagellum assembly to virulence: the extended family of type III export chaperones. Trends Microbiol. 8:202–204. 10.1016/S0966-842X(00)01751-0 10785634

[16] Aldridge P Karlinsey J Hughes KT 2003 The type III secretion chaperone FlgN regulates flagellar assembly via a negative feedback loop containing its chaperone substrates FlgK and FlgL. Mol. Microbiol. 49:1333–1345. 10.1046/j.1365-2958.2003.03637.x 12940991

[17] Thomas J Stafford GP Hughes C 2004 Docking of cytosolic chaperone-substrate complexes at the membrane ATPase during flagellar type III protein export. Proc. Natl. Acad. Sci. U. S. A. 101:3945–3950. 10.1073/pnas.0307223101 15001708 PMC374349

[18] Evans LD Stafford GP Ahmed S Fraser GM Hughes C 2006 An escort mechanism for cycling of export chaperones during flagellum assembly. Proc. Natl. Acad. Sci. U. S. A. 103:17474–17479. 10.1073/pnas.0605197103 17088562 PMC1859953

[19] Minamino T MacNab RM 2000 Interactions among components of the Salmonella flagellar export apparatus and its substrates. Mol. Microbiol. 35:1052–1064. 10.1046/j.1365-2958.2000.01771.x 10712687

[20] Minamino T Shimada M Okabe M Saijo-Hamano Y Imada K Kihara M Namba K 2010 Role of the C-terminal cytoplasmic domain of FlhA in bacterial flagellar type III protein export. J. Bacteriol. 192:1929–1936. 10.1128/JB.01328-09 20118266 PMC2838044

[21] Bange G Kummerer N Engel C Bozkurt G Wild K Sinning I 2010 FlhA provides the adaptor for coordinated delivery of late flagella building blocks to the type III secretion system. Proc. Natl. Acad. Sci. U. S. A. 107:11295–11300. 10.1073/pnas.1001383107 20534509 PMC2895114

[22] Minamino T Namba K 2008 Distinct roles of the FliI ATPase and proton motive force in bacterial flagellar protein export. Nature 451:485–488. 10.1038/nature06449 18216858

[23] Paul K Erhardt M Hirano T Blair DF Hughes KT 2008 Energy source of flagellar type III secretion. Nature 451:489–492. 10.1038/nature06497 18216859

[24] Ozin AJ Claret L Auvray F Hughes C 2003 The FliS chaperone selectively binds the disordered flagellin C-terminal D0 domain central to polymerisation. FEMS Microbiol. Lett. 219:219–224. 10.1016/S0378-1097(02)01208-9 12620624

[25] Bennett JC Thomas J Fraser GM Hughes C 2001 Substrate complexes and domain organization of the Salmonella flagellar export chaperones FlgN and FliT. Mol. Microbiol. 39:781–791. 10.1046/j.1365-2958.2001.02268.x 11169117 PMC2528293

[26] Fraser GM Bennett JC Hughes C 1999 Substrate-specific binding of hook-associated proteins by FlgN and FliT, putative chaperones for flagellum assembly. Mol. Microbiol. 32:569–580. 10.1046/j.1365-2958.1999.01372.x 10320579

[27] Pallen MJ Penn CW Chaudhuri RR 2005 Bacterial flagellar diversity in the post-genomic era. Trends Microbiol. 13:143–149. 10.1016/j.tim.2005.02.008 15817382

[28] Levine A Vannier F Absalon C Kuhn L Jackson P Scrivener E Labas V Vinh J Courtney P Garin J Seror SJ 2006 Analysis of the dynamic Bacillus subtilis Ser/Thr/Tyr phosphoproteome implicated in a wide variety of cellular processes. Proteomics 6:2157–2173. 10.1002/pmic.200500352 16493705

[29] Macek B Mijakovic I Olsen JV Gnad F Kumar C Jensen PR Mann M 2007 The serine/threonine/tyrosine phosphoproteome of the model bacterium Bacillus subtilis. Mol. Cell. Proteomics 6:697–707. 10.1074/mcp.M600464-MCP200 17218307

[30] Elsholz AK Turgay K Michalik S Hessling B Gronau K Oertel D Mader U Bernhardt J Becher D Hecker M Gerth U 2012 Global impact of protein arginine phosphorylation on the physiology of Bacillus subtilis. Proc. Natl. Acad. Sci. U. S. A. 109:7451–7456. 10.1073/pnas.1117483109 22517742 PMC3358850

[31] Jers C Pedersen MM Paspaliari DK Schutz W Johnsson C Soufi B Macek B Jensen PR Mijakovic I 2010 Bacillus subtilis BY-kinase PtkA controls enzyme activity and localization of its protein substrates. Mol. Microbiol. 77:287–299. 10.1111/j.1365-2958.2010.07227.x 20497499

[32] Garnak M Reeves HC 1979 Phosphorylation of Isocitrate dehydrogenase of Escherichia coli. Science 203:1111–1112. 10.1126/science.34215 34215

[33] Deutscher J Kuster E Bergstedt U Charrier V Hillen W 1995 Protein kinase-dependent HPr-CcpA interaction links glycolytic activity to carbon catabolite repression in Gram-positive bacteria. Mol. Microbiol. 15:1049–1053. 10.1111/j.1365-2958.1995.tb02280.x 7623661

[34] Cozy LM Phillips AM Calvo RA Bate AR Hsueh YH Bonneau R Eichenberger P Kearns DB 2012 SlrA/SinR/SlrR inhibits motility gene expression upstream of a hypersensitive and hysteretic switch at the level of sD in Bacillus subtilis. Mol. Microbiol. 83:1210–1228. 10.1111/j.1365-2958.2012.08003.x 22329926 PMC3303961

[35] Hsueh YH Cozy LM Sham LT Calvo RA Gutu AD Winkler ME Kearns DB 2011 DegU-phosphate activates expression of the anti-sigma factor FlgM in Bacillus subtilis. Mol. Microbiol. 81:1092–1108. 10.1111/j.1365-2958.2011.07755.x 21736639 PMC3193947

[36] Kutsukake K Okada T Yokoseki T Iino T 1994 Sequence analysis of the *flgA* gene and its adjacent region in Salmonella Typhimurium, and identification of another flagellar gene, *flgN*. Gene 143:49–54. 10.1016/0378-1119(94)90603-3 8200538

[37] Verhamme DT Kiley TB Stanley-Wall NR 2007 DegU co-ordinates multicellular behaviour exhibited by Bacillus subtilis. Mol. Microbiol. 65:554–568. 10.1111/j.1365-2958.2007.05810.x 17590234

[38] Kiley TB Stanley-Wall NR 2010 Post-translational control of Bacillus subtilis biofilm formation mediated by tyrosine phosphorylation. Mol. Microbiol. 78:947–963. 10.1111/j.1365-2958.2010.07382.x 20815827

[39] Arnaud M Chastanet A Debarbouille M 2004 New vector for efficient allelic replacement in naturally nontransformable, low-GC-content, gram-positive bacteria. Appl. Environ. Microbiol. 70:6887–6891. 10.1128/AEM.70.11.6887-6891.2004 15528558 PMC525206

[40] Liu H Naismith JH 2009 A simple and efficient expression and purification system using two newly constructed vectors. Protein Expr. Purif. 63:102–111. 10.1016/j.pep.2008.09.008 18845260 PMC3315830

[41] Ostrowski A Mehert A Prescott A Kiley TB Stanley-Wall NR 2011 YuaB functions synergistically with the exopolysaccharide and TasA amyloid fibers to allow biofilm formation by Bacillus subtilis. J. Bacteriol. 193:4821–4831. 10.1128/JB.00223-11 21742882 PMC3165672

[42] Sievers F Wilm A Dineen D Gibson TJ Karplus K Li W Lopez R McWilliam H Remmert M Soding J Thompson JD Higgins DG 2011 Fast, scalable generation of high-quality protein multiple sequence alignments using Clustal Omega. Mol. Syst. Biol. 7:539. 10.1038/msb.2011.75 21988835 PMC3261699

[43] Jones DT 1999 Protein secondary structure prediction based on position-specific scoring matrices. J. Mol. Biol. 292:195–202. 10.1006/jmbi.1999.3091 10493868

[44] Buchan DW Minneci F Nugent TC Bryson K Jones DT 2013 Scalable web services for the PSIPRED protein Analysis Workbench. Nucleic Acids Res. 41:W349–357. 10.1093/nar/gkt381 23748958 PMC3692098

[45] Blair KM Turner L Winkelman JT Berg HC Kearns DB 2008 A molecular clutch disables flagella in the Bacillus subtilis biofilm. Science 320:1636–1638. 10.1126/science.1157877 18566286

[46] Allan C Burel JM Moore J Blackburn C Linkert M Loynton S Macdonald D Moore WJ Neves C Patterson A Porter M Tarkowska A Loranger B Avondo J Lagerstedt I Lianas L Leo S Hands K Hay RT Patwardhan A Best C Kleywegt GJ Zanetti G Swedlow JR 2012 OMERO: flexible, model-driven data management for experimental biology. Nat. Methods 9:245–253. 10.1038/nmeth.1896 22373911 PMC3437820

[47] Murray EJ Strauch MA Stanley-Wall NR 2009 σX is involved in controlling Bacillus subtilis biofilm architecture through the AbrB homologue Abh. J. Bacteriol. 191:6822–6832. 10.1128/JB.00618-09 19767430 PMC2772499

[48] Karlinsey JE Tanaka S Bettenworth V Yamaguchi S Boos W Aizawa SI Hughes KT 2000 Completion of the hook-basal body complex of the Salmonella Typhimurium flagellum is coupled to FlgM secretion and *fliC* transcription. Mol. Microbiol. 37:1220–1231. 10.1046/j.1365-2958.2000.02081.x 10972838

[49] Murray EJ Kiley TB Stanley-Wall NR 2009 A pivotal role for the response regulator DegU in controlling multicellular behaviour. Microbiology 155:1–8. 10.1099/mic.0.023903-0 19118340

[50] Miller J 1972 Experiments in molecular genetics. Cold Spring Harbor Laboratory Press, Cold Spring Harbor, NY

[51] Aizawa SI Dean GE Jones CJ Macnab RM Yamaguchi S 1985 Purification and characterization of the flagellar hook-basal body complex of Salmonella Typhimurium. J. Bacteriol. 161:836–849 2982790 10.1128/jb.161.3.836-849.1985 PMC214974

[52] Kubori T Okumura M Kobayashi N Nakamura D Iwakura M Aizawa SI 1997 Purification and characterization of the flagellar hook-basal body complex of Bacillus subtilis. Mol. Microbiol. 24:399–410. 10.1046/j.1365-2958.1997.3341714.x 9159525

[53] Gygi D Fraser G Dufour A Hughes C 1997 A motile but non-swarming mutant of Proteus mirabilis lacks FlgN, a facilitator of flagella filament assembly. Mol. Microbiol. 25:597–604. 10.1046/j.1365-2958.1997.5021862.x 9302021

[54] Fraser GM Hughes C 1999 Swarming motility. Curr. Opin. Microbiol. 2:630–635. 10.1016/S1369-5274(99)00033-8 10607626

[55] Mukherjee S Yakhnin H Kysela D Sokoloski J Babitzke P Kearns DB 2011 CsrA-FliW interaction governs flagellin homeostasis and a checkpoint on flagellar morphogenesis in Bacillus subtilis. Mol. Microbiol. 82:447–461. 10.1111/j.1365-2958.2011.07822.x 21895793 PMC3192257

[56] Diethmaier C Pietack N Gunka K Wrede C Lehnik-Habrink M Herzberg C Hubner S Stulke J 2011 A novel factor controlling bistability in Bacillus subtilis: the YmdB protein affects flagellin expression and biofilm formation. J. Bacteriol. 193:5997–6007. 10.1128/JB.05360-11 21856853 PMC3194898

[57] Chadsey MS Karlinsey JE Hughes KT 1998 The flagellar anti-sigma factor FlgM actively dissociates Salmonella Typhimurium sigma28 RNA polymerase holoenzyme. Genes Dev. 12:3123–3136. 10.1101/gad.12.19.3123 9765212 PMC317189

[58] Kutsukake K 1994 Excretion of the anti-sigma factor through a flagellar substructure couples flagellar gene expression with flagellar assembly in Salmonella Typhimurium. Mol. Gen. Genet. 243:605–612 8028576 10.1007/BF00279569

[59] Karlinsey JE Lonner J Brown KL Hughes KT 2000 Translation/secretion coupling by type III secretion systems. Cell 102:487–497. 10.1016/S0092-8674(00)00053-2 10966110

[60] Yakhnin H Pandit P Petty TJ Baker CS Romeo T Babitzke P 2007 CsrA of Bacillus subtilis regulates translation initiation of the gene encoding the flagellin protein (*hag*) by blocking ribosome binding. Mol. Microbiol. 64:1605–1620. 10.1111/j.1365-2958.2007.05765.x 17555441

[61] Gerwig J Kiley TB Gunka K Stanley-Wall N Stulke J 2014 The protein tyrosine kinases EpsB and PtkA differentially affect biofilm formation in Bacillus subtilis. Microbiology 160:682–691. 10.1099/mic.0.074971-0 24493247 PMC3973450

[62] Petranovic D Michelsen O Zahradka K Silva C Petranovic M Jensen PR Mijakovic I 2007 Bacillus subtilis strain deficient for the protein-tyrosine kinase PtkA exhibits impaired DNA replication. Mol. Microbiol. 63:1797–1805. 10.1111/j.1365-2958.2007.05625.x 17367396

[63] Mijakovic I Poncet S Boel G Maze A Gillet S Jamet E Decottignies P Grangeasse C Doublet P Le Marechal P Deutscher J 2003 Transmembrane modulator-dependent bacterial tyrosine kinase activates UDP-glucose dehydrogenases. EMBO J. 22:4709–4718. 10.1093/emboj/cdg458 12970183 PMC212725

[64] Fuhrmann J Schmidt A Spiess S Lehner A Turgay K Mechtler K Charpentier E Clausen T 2009 McsB is a protein arginine kinase that phosphorylates and inhibits the heat-shock regulator CtsR. Science 324:1323–1327. 10.1126/science.1170088 19498169

[65] Landry CR Levy ED Michnick SW 2009 Weak functional constraints on phosphoproteomes. Trends Genet. 25:193–197. 10.1016/j.tig.2009.03.003 19349092

[66] Beltrao P Albanese V Kenner LR Swaney DL Burlingame A Villen J Lim WA Fraser JS Frydman J Krogan NJ 2012 Systematic functional prioritization of protein posttranslational modifications. Cell 150:413–425. 10.1016/j.cell.2012.05.036 22817900 PMC3404735

[67] Sourjik V Schmitt R 1998 Phosphotransfer between CheA, CheY1, and CheY2 in the chemotaxis signal transduction chain of Rhizobium meliloti. Biochemistry 37:2327–2335. 10.1021/bi972330a 9485379

