# Supplementary material for: FlgN Is Required for Flagellum-Based Motility by Bacillus subtilis
Source: J Bacteriol. 2014 Jun;196(12):2216–26. doi: 10.1128/JB.01599-14 (PMC4054197; doi:10.1128/JB.01599-14)
Supplement: Supplemental material [file supp_196_12_2216__index.html]

FlgN Is Required for Flagellum-Based Motility by Bacillus subtilis — Supplemental material 

# FlgN Is Required for Flagellum-Based Motility by Bacillus subtilis

## Supplemental material

**Files in this Data Supplement:**

- Supplemental file 1 -

  Table S1, strains

  Table S2, plasmids

  Table S3, primers

  Table S4, mass spectrometry analysis of top 12 proteins identified from hook-basal body enrichment of NCIB3610

  Supplemental methods

  Fig. S1, amino acids identified in mass spectrometry identification of Hag

  Fig. S2, Western blot analysis of FlgE

  Fig. S3, characterization of His-tagged FlgK strains and enrichment of hook-basal bodies

  PDF, 708K
