# Supplementary material for: FlgN Is Required for Flagellum-Based Motility by Bacillus subtilis
Source: J Bacteriol. 2014 Jun;196(12):2216–26. doi: 10.1128/JB.01599-14 (PMC4054197; doi:10.1128/JB.01599-14)
Supplement: Supplemental material [file JB.01599-14_zjb999093185so1.pdf]

Supplemental Information.

FlgN function in flagellar based motility by *Bacillus subtilis*

Lynne S. Cairns [1], Victoria L. Marlow [1], Taryn B. Kiley [1], Christopher Birchall [2], Adam Ostrowski [1], Phillip D. Aldridge [2] and Nicola R. Stanley-Wall [1]

[1.] Division of Molecular Microbiology, College of Life Sciences, University of Dundee, Dundee, UK DD1 5EH

[2.] Centre for Bacterial Cell Biology, Medical Sciences New Building, Newcastle University, Richardson Road, Newcastle upon Tyne, UK

*Running Title: The function of FlgN in *Bacillus subtilis*.

To whom correspondence should be addressed:

Nicola R. Stanley-Wall

Division of Molecular Microbiology, College of Life Sciences, University of Dundee, Dundee DD1 5EH.

Email: n.r.stanleywall@dundee.ac.uk Tel: +44(0)1382 385136; Fax: +44(0)1382 388216;

Figure S1 Amino Acids Identified in Mass Spectrometry Identification of Hag. The protein sequence of Hag is shown with amino acids from peptide sequences which were identified by mass spectrometry analysis in the identification of Hag in whole cell lysates highlighted in bold.

Figure S2 Western Blot Analysis of FlgE. Western blot analysis of cellular (including assembled flagella) fractions of the wild-type (NCIB3610), Δ*flgE* (NRS4042), Δ*hag* (DS1677), Δ*fliD* (NRS4041), Δ*flgN* (NRS3570) and Δ*flgK-flgL* (NRS4060) probed with the α-FlgE antibody.

Figure S3 Characterisation of His-tagged FlgK Strains and Enrichment of Hook-Basal Bodies. (A) swimming expansion assay of wild-type (NCIB3610), Δ*flgK-flgL* (NRS4060), His-*flgK* (NRS4812) and *flgK*-His (NRS4801). Photographs were taken after 24 hours incubation at room temperature. **(B)** Coomassie stained SDS-PAGE gel analysis of flagellar hook-basal body proteins enriched from the wild-type strain (NCIB3610). Mass spectrometry analysis of this sample is detailed in Table S4.

Table S1 Strains used in this study.

Strain	Relevant Genotype [a]	Source [b]
**NCIB3610**	Prototroph	B.G.S.C.
**168**	*trpC2*	(1)
**NRS1314**	3610 *degU::pBL204 (cml)*	(2)
**DS1677**	3610 *Δhag*	Kind gift D. Kearns
**NRS1467**	3610 *amyE::Phy-spank-gfp-lacI (spc)*	(2)
**NRS2544**	3610 *ΔptkA*	(3)
**NRS3070**	168 *sacA::Phag-yfp (kan)*	pNW546 →168
**NRS3076**	3610 *sacA::Phag-yfp (kan)*	SPP1 3070 →3610
**NRS3256**	168 *amyE::*$P_{hy\text{-}spank}$*-flgN-lacI (spc)*	pNW380 →3610
**NRS3570**	3610 *ΔflgN*	pNW399 →3610
**NRS3571**	3610 *flgN* $Y^{49}A$	pNW801 →3610
**NRS3578**	3610 *ΔflgN+ amyE::*$P_{hy\text{-}spank}$*-flgN-lacI (spc)*	SPP1 3256 →NRS3570
**NRS3708**	3610 *ΔflgN + sacA::*P_{hag}*-yfp (kan)*	SPP1 3070 →NRS3570
**NRS3713**	3610 *ΔflgN + sacA::*P_{hag}*-yfp (kan) + amyE::*$P_{hy\text{-}spank}$*-flgN-lacI (spc)*	SPP1 3256 →NRS3708
**NRS3718**	3610 *ΔflgN hag* $T^{209}C$	pNW1010 →NRS3570
**NRS3719**	3610 *hag* $T^{209}C$	pNW1010 →3610
**NRS3724**	3610 *flgN* $R^{49}E$	pNW1012 →3610
**NRS3735**	168 *amyE::*$P_{hy\text{-}spank}$*-flgKL-lacI (spc)*	pNW1017 →168
**NRS4017**	3610 *flgN* $R^{60}E$	pNW1028 →3610
**NRS4041**	3610 *ΔfliD*	pNW1034 →3610
**NRS4042**	3610 *ΔflgE*	pNW1036 →3610
**NRS4043**	3610 *ΔflgN+ amyE::*$P_{hy\text{-}spank}$*-flgKL-lacI (spc)*	SPP1 3735 →NRS3570
**NRS4060**	3610 *ΔflgKL*	pNW1042 →3610
**NRS4063**	3610 *flgN* $R^{60}A$	pNW1027 →3610
**NRS4064**	3610 *ΔflgKL + amyE::*$P_{hy\text{-}spank}$*-flgKL-lacI (spc)*	SPP1 3735 → NRS4060
**NRS4071**	3610 *ΔflgKL+ sacA::*P_{hag}*-yfp (kan)*	pNW1042 →NRS4060
**NRS4078**	3610 *ΔflgKL + sacA::*P_{hag}*-yfp (kan) + amyE::*$P_{hy\text{-}spank}$*-flgKL-lacI (spc)*	SPP1 3735 → NRS4078
**NRS4794**	168 *amyE::*P_{hag}*-lacZ* translational *(cml)*	pNW1069 →168
**NRS4795**	3610 *amyE::*P_{hag}*-lacZ* translational *(cml)*	SPP1 4794 →3610
**NRS4796**	3610 *ΔflgN + amyE::*P_{hag}*-lacZ* translational *(cml)*	SPP1 4794 →3570
**NRS4798**	3610 *ΔflgE + amyE::*P_{hag}*-lacZ* translational *(cml)*	SPP1 4794 →4042
**NRS4799**	3610 *ΔflgKL + amyE::*P_{hag}*-lacZ* translational *(cml)*	SPP1 4794 →4060
**NRS4801**	3610 *His-flgK*	pNW1063 →3610
**NRS4812**	3610 *flgK-His*	pNW1065 →3610

[a] Drug resistance cassettes are indicated as follows: *kan*, kanamycin resistance; *mls*, lincomycin/erythromycin resistance; *cml*, chloramphenicol resistance; and *spc*, spectinomycin resistance.

[b] The direction of strain construction is indicated with DNA or phage (SPP1) (→) recipient strain. B.S.G.C. is the *Bacillus* genetic stock centre.

Table S2. Plasmids used in this study.

Plasmid	Relevant Genotype [a]	Source
**pDR111**	*amyE* integration plasmid (spc)	(4)
**pBL132**	*cml*^{R} cassette in pUC19	(5)
**pBL165**	*amyE* integration plasmid $P_{spac\text{-}hy}$-*gfp mut2*	(5)
**pDR183**	*lacA integration plasmid*	(5)
**pKM3a**	*yfp amyE* integration plasmid	Kind gift D. Rudner
**pSAC-KAN**	*sacA* integration plasmid	(6)
**pEHISGFPTEV**	Protein expression vector	(7)
**pDG1728**	*lacZ* reporter fusion plasmid, *amyE* integration	(8)
**pMAD**	In-frame markerless mutation plasmid	(9)
**pNW380**	*flgN* coding region in pDR111	This study
**pNW398**	*flgN* in pUC19	This study
**pNW399**	Δ*flgN* in pMAD	This study
**pNW546**	P_{hag}-*yfp* in pSAC-KAN	This study
**pNW642**	*sigA* in pEHISGFPTEV	This study
**pNW700a**	P_{hag} in pKM3a	This study
**pNW800**	*flgN* Y^{49}A in pUC19	This study
**pNW801**	*flgN* Y^{49}A in pMAD	This study
**pNW1006**	*hag* in pBL132	This study
**pNW1009**	*hag* T^{209}C in pBL132	This study
**pNW1010**	*hag* T^{209}C in pMAD	This study
**pNW1011**	*flgN* Y^{49}E in pUC19	This study
**pNW1012**	*flgN* Y^{49}E in pMAD	This study
**pNW1017**	*flgKL* coding region in pDR111	This study
**pNW1025**	*flgN* R^{60}A in pUC19	This study
**pNW1026**	*flgN* R^{60}E in pUC19	This study
**pNW1027**	*flgN* R^{60}A in pMAD	This study
**pNW1028**	*flgN* R^{60}E in pMAD	This study
**pNW1034**	Δ*fliD* in pMAD	This study
**pNW1036**	Δ*flgE* in pMAD	This study
**pNW1042**	Δ*flgKL* in pMAD	This study
**pNW1063**	His-flgK in pMAD	This study
**pNW1065**	*flgK*-His in pMAD	This study
**pNW1069**	P_{hag} in pDG1728	This study

[a] Drug resistance cassettes are indicated as follows: *cml*, chloramphenicol resistance and *spc*, spectinomycin resistance.

Table S3 Primers used in this study.

Primer	Target	Sequence [a]	Position [b]
**DEN5**	*rRNA*	TCACGRCACGAGCTGACGAC	Internal 16S rRNA
**DEN7**	*rRNA*	ACTCCTACGGGAGGCAGC	Internal 16S rRNA
**NSW652**	*hag*	CCGGAATTCGAATTGACGCCCCAAAGCATATTG	-272→-248
**NSW653**	*hag*	GGCAAGCTTCTGAATATGTTGTTAAGGCACGTC	-38→-14
**NSW860**	*sigA*	GCATCCATGGCTGATAAACAAACCCACG	+5→+22
**NSW861**	*sigA*	GCATCTCGAGTTATTCAAGGAAATCTTTC	+1095→+1116
**NSW936**	*flgN*	GCATGGATCCATC AGG CAG AAG CGA ATT CAG	-588→-558
**NSW937**	*flgN*	GCATTCTAGATAATTGCCTTCGCTGACATGG	+92→+123
**NSW938**	*flgN*	GCATTCTAGACTGTTTGAT TCAAAAGCTTAGCAGAAAG	+453→+490
**NSW939**	*flgN*	GCATGTCGACCAACACAGTTGTATTTAGCT TGC	+1104→1137
**NSW940**	*flgN*	GCAT GTCGACAAGCAATAAAAAAGGAGAAAGCCC	-34→-1
**NSW941**	*flgN*	GCATGCATGCAATTCCTTTCTGCTAAGCTTTTGAATC	+469→+505
**NSW942**	*flgN*	ACAAAAGAGCAAAAA**GCA**ATTCAAGCAATCACG	+130→+162
**NSW943**	*flgN*	CGTGATTGCTTGAAT**TGC**TTTTTGCTCTTTTGT	+130→+162
**NSW1401**	*hag*	GCATGGATCCGCGATCTCTGAAAA	+39→+52
**NSW1402**	*hag*	CGATGGATCCTCGTTTATATCGACTAAGT	+1103→1121
**NSW1403**	*hag*	GCAGATAATGCAGCAGATT**TG**TGCTGATATCGGTTTCGATGC	+107→+147
**NSW1404**	*hag*	GCATCGAAACCGATATCAGC**AC**AATCTGCTGCATTATCTGC	+107→+147
**NSW1436**	*flgN*	ACAAAAGAGCAAAAA**GAA**ATTCAAGCAATCACG	+130→+162
**NSW1437**	*flgN*	CGTGATTGCTTGAAT**TTC**TTTTTGCTCTTTTGT	+130→+162
**NSW1438**	*yvyF*	GAAAACCGGCAATCAACTTTGAGC	+133→+156
**NSW1439**	*yvyF*	CTCGTGGTTCAAATGATCCATTTGATC	+322→+348
**NSW1440**	*flgM*	ATCAATCAATTTGGAACACAATCCG	+7→+31
**NSW1441**	*flgM*	GTCTACTTTGTATGACCCGTTTTC	+196→+216
**NSW1442**	*flgN*	GCC GGC AAAACAAAAGAGCTTTCT	+97→+120
**NSW1443**	*flgN*	TCCGAGAACTTGAGAAAGAGATTCGTA	+283→+309
**NSW1444**	*flgK*	TACAACAAATCAGGCGGGAATGCA	+682→+705
**NSW1445**	*flgK*	AGACTCTATAAACCCTAAAAGGGATCCC	+894→+921
**NSW1446**	*flgL*	CAAGCGATTGGCGTAGAGGTAAA	+322→+344
**NSW1447**	*flgL*	GCCTCCAAAAGCTGACTTTGGATC	+526→+549
**NSW1459**	*yviE*	CTGCTCATTTTTAAGCTGGC	+64→+83
**NSW1462**	*flgK*	GCATGTCGACTTAGCAGAAAGGAATT	-22→-6
**NSW1463**	*flgL*	GACTGCATGCTTACTTTAAAAAGTCAAT	+880→+897
**NSW1464**	*flgN*	CAGACAGAAGATGAC**GCG**ATCAAAACAACTTCG	+163→+193
**NSW1465**	*flgN*	CGAAGTTGTTTTGAT**CGC**GTCATCTTCTGTCTG	+163→+193
**NSW1466**	*flgN*	CAGACAGAAGATGAC**GAG**ATCAAAACAACTTCG	+163→+193
**NSW1467**	*flgN*	CGAAGTTGTTTTGAT**CTC**GTCATCTTCTGTCTG	+163→+193
**NSW1670**	*hag*	GCAGGATCCAAGAAGAACAAATCATTCTTTTTGAAAG	-766→-727
**NSW1671**	*hag*	TTGCGGTCGACGTGGTTAATTCTCATTGTTTTGTTCCT	-12→+25

[a] Underlined sequences indicate endonuclease restriction cut sites. Bold sequences represent base pairs mutated by site-directed mutagenesis.

[b] Position of primer is indicated in relation to the translational start site (noted as +1) of the named gene.

Table S4. Mass spectrometry analysis of top 12 proteins identified from hook-basal body enrichment of NCIB3610. The top 12 proteins from the NCIB3610 sample are presented as identified by their Mascot protein score.

**Protein Rank**	**Protein Description**	**Mascot Protein Score**	**Coverage (%)**	**Unique Peptides**	**No. Peptides**
1	flagellin [Bacillus subtilis subsp. subtilis str. 168]	13717.54	96.71	38	45
2	flagellar basal body rod protein FlgG [Bacillus subtilis subsp. subtilis str. 168]	6251.24	96.97	27	27
3	elongation factor Tu [Bacillus subtilis subsp. subtilis str. 168]	6107.34	92.42	38	38
4	surfactin synthetase [Bacillus subtilis subsp. subtilis str. 168	3582.23	34.80	84	104
5	surfactin synthetase [Bacillus subtilis subsp. subtilis str. 168]	3267.57	38.44	77	97
6	flagellar MS-ring protein [Bacillus subtilis subsp. subtilis str. 168]	3254.30	70.34	32	32
7	flagellar hook-associated protein FlgK [Bacillus subtilis subsp. subtilis str. 168	2784.52	73.77	27	27
8	putative flagellin [Bacillus subtilis subsp. subtilis str. 168]	2249.41	48.75	1	11
9	glyceraldehyde-3-phosphate dehydrogenase [Bacillus subtilis subsp. subtilis str. 168	1957.55	54.63	15	15
10	30S ribosomal protein S2 [Bacillus subtilis subsp. subtilis str. 168]	1776.43	84.55	26	26
11	flagellar capping protein [Bacillus subtilis subsp. subtilis str. 168]	1765.60	63.25	29	29
12	flagellar hook-associated protein FlgL [Bacillus subtilis subsp. subtilis str. 168]	1693.66	67.45	16	16

Plasmid Construction.

Construction of Plasmid pNW546. Plasmid pNW546 was constructed to assay *hag* transcription using YFP as a reporter. The promoter region of *hag* was PCR amplified from *B. subtilis* 3610 chromosomal DNA using primers NSW652 and NSW653 and ligated into pKM3a using EcoRI and HindIII restriction sites to create pNW700a. pNW700a was digested with EcoRI and BamHI to release the P*hag-yfp* fragment which was ligated into pSAC-KAN (6), to allow for integration at the non-essential *sacA* locus.

Construction of Plasmid pNW1017. Plasmid pNW1017, used to introduce the *flgK* and *flgL* coding regions under the control of the IPTG-inducible promoter $P_{hy\text{-}spank}$ at the non-essential *amyE* locus, is a derivative of pDR111 (4). The coding regions of *flgK* and *flgL* (including ribosome binding site) and the intergenic region were amplified from *B. subtilis* 3610 chromosomal DNA with primers NSW1462 and NSW1463. The PCR product was digested SalI/SphI and cloned into pDR111 cut the same. Plasmid pNW380 for *flgN* was constructed in an identical manner using primers NSW940 and NSW941.

Construction of Plasmid pNW1069. Plasmid pNW1069 was constructed to introduce a *hag'-'lacZ* translational reporter fusion at the non-essential *amyE* locus. The *hag* promoter, the *hag* 5'UTR and the 5' end of the *hag* open reading frame (10) were amplified by PCR with the primers NSW1670 and NSW1671 from *B. subtilis* 3610 chromosomal DNA. The PCR product was digested BamHI/SalI and cloned into the BamHI and SalI sites of pDG1728 (8).

Construction of Plasmid pNW1034. Plasmid pNW1034 was constructed to introduce an in-frame markerless deletion of *fliD* to the chromosome. A region of approximately 1000 bp, including 500bp upstream of *fliD*, the first two codons and final two codons of *fliD*, and 500 bp downstream of *fliD* was synthesised and cloned into a pUC cloning plasmid by Dundee Cell Products (http://www.dundeecellproducts.com/). The Δ*fliD* region was excised from the pUC plasmid with EcoRI and BamHI and cloned into the EcoRI and BamHI sites of pMAD (9). Plasmid pNW1036 and pNW1042 were constructed in a similar manner. Synthesised sequences are detailed below. Restriction sites are underlined and the first two codons and final two codons of each gene deleted are highlighted in bold.

Construction of pNW1063. Plasmid pNW1063 was constructed to introduce a poly-Histidine epitope tag at the N-terminus of *flgK*. A region of approximately 1000 bp including 500bp upstream of the *flgK* transcriptional start site, the *flgK* start codon, a region encoding 10 Histidine residues and 500bp downstream of the *flgK* transcriptional start site was synthesised and cloned into a pUC cloning

plasmid by GenScript (http://www.genscript.com/). The His-*flgK* region was excised from the pUC plasmid with BamHI and cloned into the BamHI site of pMAD (9). pNW1065 was constructed in a similar manner. Synthesised sequences are detailed below. Restriction sites are underlined and His tag regions highlighted in bold underline.

Regions of DNA synthesised for cloning into pMAD.

Δ*fliD* used to construct pNW1034

GAATTCACAAGAAATCTAAAACAGAAGATTTTTTTCCAAAAATATGTGTAATCTTATCTCGACTTAGT
CGATATAAACGATAGATTGGGGCATAGGGGATGATCAATTGAACATTGAAAGGCTCACTACGTTACAA
CCTGTTTGGGATCGTTATGATACTCAAATACATAATCAGAAAGATAATGATAACGAGGTTCCTGTTCA
TCAAGTTTCATATACCAATCTTGCTGAAATGGTGGGGAAATGAACAAGCTTTTGGAACCTTCGCAAG
TTCATCTGAAGTTCGAGCTTCATGACAAGTTAAATGAATACTATGTAAAGGTAATAGAGGACTCTACA
AATGAAGTGATCCGCGAAATTCCACCAAAACGGTGGCTTGATTTTTATGCGGCTATGACTGAATTTCT
TGGGTTATTTGTAGATGAAAAAAAGTAGAATAGGAGTGGTTTGAG**ATGGTCCAATAA**ATGTAATTTGG
AGGATGACACATGGCGATCCAAAATCCATATACAGCCTATCAGCAAAATTCAGTGAATACGGCTACAC
CCGGGGAGCTGACGCTTATGCTGTATAATGGCTGTTTGAAATTTATAAGACTTGCCGCTCAGGCCATT
GAGAATGATGATATGGAACGTAAAAATGAAAATCTGATTAAAGCGCAAAATATTATTCAGGAATTAAA
TTTTACACTTAACCGTAACATAGAGCTTTCCGCTTCTATGGGTGCGATGTACGATTATATGTATCGCA
GATTGGTACAGGCAAATATCAAAAATGATACGGGCATGCTGGCTGAGGTTGAAGGTTATGTAACAGAT
TTTCGCGATGCTTGGAAACAAGCCATTCAAAGTGAGCGGAAGGACCGGCACGGATCAGGCGGGATCGC
ATGAATAATATAGATCAACTATACACTGAGACGAAGAGTATGCTGTCACACATACAAAATACGCCGGA
AAGCGATGAACTTTTAAAGCAAATTGAAGACTTTGTGGCTACACGGTCTGAACTGATTCAGGAGATAT
CTCTGCCGCTTTCAGGATCC

Δ*flgE* used to construct pNW1036

GAATTCATGACTTCTATAAGTTCAGAATATAAACTGCCTGAAAAAACGAACACTGTGTCGACGAACAA
CAGCAGCTTGGGGAAAGACGAGTTTTTAAAAATATTAATGACTCAAGTTCAAAACCAAGATCCGCTTA
ACCCGATTGACGATAAAGAATTTATCAGCCAGATGGCGACTTTTTCAAGCTTGGAGCAAATGATGAAT
CTGAATACGACAATGACTCAATTCGTTGAAAACCAAGATCCGTTTACAACGTATGTTGATTGGATGGG
AAAAGAAGTATCTTGGACTGATGGTAAAAGTGCAACAGATAAAACAGGCACAGTAAGCTCTGTTAAAC
ATTTTAAAGGAAATTATTATCTCGTTCTTGATGATGGGACCGAGATCAGTCCTGCGAATGTCATGTCT
GTGGGACAATCATCTAAATAAAAACATCTGGGGGAATATATT**ATGTTACGTTAA**GGAGGGAGGGGAGG
CGAGTAATCGCTTCTCCGCTGTTTTATGATTAAAGTAACCCGTTTGAACGGGCAGCCCTTTACACTGA
ATGCGCTATTTATTGAACAGATTGAATGTTTTCCGGATACTACAATTACTCTGTCAAATGGTAAGAAG
TTTGTAGTAAAAGAAGATGAAGAAGCTGTTCTGGAAAAGATCGCAGCTTTCTACCGAAAAATACAAAT
ATTTGCAATGGATCAAGGAATAGAGGAACCGGAATGAAGAAAAAGTTAATGATCATATTACTAATTAT
TCTTATCGTAATTGGTGCTCTCGGGGCGGCGGCTTATTTTGTTTTAGGCGGAAAGTCCGAAAAAAGTG
AAGCGAAAAAAAGTATTGATGAAATCGTTGCGTCTTCTGTTGATGTAGAAGAGATCACAACAAATTTA
AAGTCTGATAACATTATCCGTCTTGCTATTAAACTTGAAACTGATTCTGATAAATCAAAAGAAGAACT
TGAGAAACGTGATTTCCAAGTGAAAGACGCTGTTATATCACTGCTGGCTGATACGAATGCTGGGATCC

Δ*flgKL* used to construct pNW1042

GGATCCTGTCAGCGAAGGCAATTATTGAACAATTGAAGCGACTTTGCGTTCTGCATGAGCACCTGCTC
ACGCTGTCTGAAGAAAAGACGGAAGCGCTCAAAGCCGGCAAAACAAAAGAGCTTTCTAACATTTTGAC
AAAAGAGCAAAAAATATATTCAAGCAATCACGCAGACAGAAGATGACCGGATCAAAACAACTTCGGCCT

TTCTCGGATATAGCGAAAATAATACTATTTCCGCATGTATCGCCAAAACCTCAGGCAGTGAAAAGGAAGAGCTGGAACAACTATACGAATCTCTTTCTCAAGTTCTCGGACGTCTGAAAAAAGTAAATGAGATGAATAGGCAGCTGACAAGAGACGCGCTGCAATTCATCTCTATTTCGTACGATATGCTGGTTCCTAAGGAAAATAACTTCAATTACAGCAAATCAATTAAAGCTGAGCTGCCGAAAAGTAGCAAAATGAAACTGTTTGATTCAAAAGCTTAGCAGAAAGGAATTCAGAAA**ATGACAAAGTAA**GCGGCTCTTAGGAGTTCGCTTTTTTTATAGTTCAGGAGGTAGAGTGATGCAGATTCCCAGATTGATTATGCATAGTGTTCAAGGAAAAATTGGTTTAACAACGACGCCTGCCAGCTTAAAAATGGAGCAGCCTCAAGCTGATCTAGAGATCGAACAGCCGAGTGCGGAAATGGAAATATCGGTGACACCTGGAAAACTCACGATTGACCAGACACAAGCATGGGAAGAATTAGACAGAAAGCATGTTTTCAAGAGAATTGAAGAAGCCGCCCAACAAGGGCATGAGGATGTAATGGAGGGAATAGCACGCACTGCAGAAGAAGGCGACGAGCTTATGAAGATTGAAAATAAGGGGAACCCAATCGCTTCACAAGCAAGGAGGAACTCTGAAATGCACCAAATTCAATTAGGCGAAAATTATGCTCCTTCTCTTTCGAGGGTGAAAATACAATATACTCCGTCACAGCTTGATGTGCAGATTACGCCGCGAAAGCCTGGGATCC

His-flgK used to construct pNW1063

GGATCCCCATGTCAGCGAAGGCAATTATTGAACAATTGAAGCGACTTTGCGTTCTGCATGAGCACCTGCTCACGCTGTCTGAAGAAAAGACGGAAGCGCTCAAAGCCGGCAAAACAAAAGAGCTTTCTAACATTTTGACAAAAGAGCAAAAATATATTCAAGCAATCACGCAGACAGAAGATGACCGGATCAAAACAACTTCGGCCTTTCTCGGATATAGCGAAAATAATACTATTTCCGCATGTATCGCCAAAACCTCAGGCAGTGAAAAGGAAGAGCTGGAACAACTATACGAATCTCTTTCTCAAGTTCTCGGACGTCTGAAAAAAGTAAATGAGATGAATAGGCAGCTGACAAGAGACGCGCTGCAATTCATCTCTATTTCGTACGATATGCTGGTTCCTAAGGAAAATAACTTCAATTACAGCAAATCAATTAAAGCTGAGCTGCCGAAAAGTAGCAAAATGAAACTGTTTGATTCAAAAGCTTAGCAGAAAGGAATTCAGAAAATG**CATCACCATCACCATCACCATCACCATCAC**ACATCTACCTTTATGGGGCTTGAAACTGCAAGGCGGGCGTTAAGCGCTCAGCAGGCAGCGTTAAGCACTACTGCAAATAACGTGGCAAATGCCAATACTGATGGTTATACAAGACAGCGGGTCTCATTGGAGGCAACTGACTATTTCCCTGCTGTATCTAAAAATGCAGAAAAAACAGCGGGACAAATGGGTACGGGCGTTCAAGGAAAATCAGTTGAGAGAATAAGAGATATCTTTCTTGACTACCAATACCGTCTTCAAAACAACAGTGCCGGATACTATGACACGAAGGCAAAAGCGCTGTCCCAAATGGAAGGCGTTTTAAATGAAACGGATGACAGCGGCTTGAACAGTGTGCTCAATTCGTTTTGGAATTCCCTGCAGGAATTATCGAATAATACAAATGAAGAAAGTGCACGTTCTGTTGTTGCTCGAAAAGGACAAGCTGTAGCTGAAACGTTTAATTATATTTCTGAATCACTTACAAATGTCCGGATCC

flgK-His used to construct pNW1065

GAATTCGAAGTGCACAGAAATGGTGTGACCAAGAGCGGTGAACAAGGCGGAGACTTTTTTGATTTTACTGGCGGTGAAACTGAACCTGCCAAGGGCGCGGCGGGCAAGATCAAAGTGGCTGACAGCATAATAGATTCAAAAGGCGCAAACATTGCTTTCTCACTGACTGGCGCAGCCAACGATAACGCAAATGCTACAAAATTAGCAAATGTTTTAACCGGTAAAATAACCATTAACGGTAAAGAAACTAGTGTTTTAGATTATTATGCGGGTCTGATTGGCGAGCTAGGGATCGAAGCTCAAGAGGCTAATCGACTGGCGTCTAATACAGAAACACAGCTGAATGATGCTGACATAAACCGTCAGCAAATGAGCGCAGTTTCTTTAGACGAAGAAATGACGAATATGATTCAATTCCAACACGCATACAATGCAGCTGCAAGAATGGTGACTTTACAAGACGAATTGCTTGATAAAGTGATCAACGGCATGGGTGTTGGAGGAAGGGGAGCAGGA**CATCACCATCACCATCACCATCACCATCAC**TAGTGGGTGTTGGAGGAAGGGTAGAGTCACATGAGAGTAACACAAGGCATGATACAGCAAAACTCACTGAGATATATCGGTTCAAGCTACTCGAAGCTGGATAAACTCCAGTCGCAGATTTCTTCAGGAAAAAAAATCTCAAAAGCTTCCGACGATCCTGTAGTAGCAATGAAAAGCTTAAAGTATAATACGCAACTGTCTCAAGTGCAGCAGTACAAAAGCAATGCTTCTCAAGCCTTTACCTGGCTCGAAAACACAGAAACAAACATTACAGAAGGAATTGACATCTTGTCAAAGGTCAGAGAATTAGCGGTTGAAGCTCAAAATGATACAAACGGCGAGCCGGAGCGGCAAGCGATTGGCGTAGAGGTAAAGCAGTTAAAGGAACAGCTTTTAAATATTGCGA

ATACACAAGTGAACGGCAGATATATCTTTAATGGCACAAATTCAGATAAGCCTCCGGTTACAGATAAC
GGAGACGGAACTTATAC<u>GGATCC</u>

References.

1. **Perego M, Hoch JA.** 1988. Sequence analysis and regulation of the *hpr* locus, a regulatory gene for protease production and sporulation in *Bacillus subtilis*. J Bacteriol **170:**2560-2567.
2. **Verhamme DT, Kiley TB, Stanley-Wall NR.** 2007. DegU co-ordinates multicellular behaviour exhibited by *Bacillus subtilis*. Mol Microbiol **65:**554-568.
3. **Kiley TB, Stanley-Wall NR.** 2010. Post-translational control of *Bacillus subtilis* biofilm formation mediated by tyrosine phosphorylation. Mol Microbiol **78:**947-963.
4. **Britton RA, Eichenberger P, Gonzalez-Pastor JE, Fawcett P, Monson R, Losick R, Grossman AD.** 2002. Genome-wide analysis of the stationary-phase sigma factor (Sigma-H) regulon of *Bacillus subtilis*. Journal of Bacteriology **184:**4881-4890.
5. **Stanley NR, Britton RA, Grossman AD, Lazazzera BA.** 2003. Identification of catabolite repression as a physiological regulator of biofilm formation by *Bacillus subtilis* by use of DNA microarrays. J Bacteriol **185:**1951-1957.
6. **Middleton R, Hofmeister A.** 2004. New shuttle vectors for ectopic insertion of genes into *Bacillus subtilis*. Plasmid **51:**238-245.
7. **Liu H, Naismith JH.** 2009. A simple and efficient expression and purification system using two newly constructed vectors. Protein Expr Purif **63:**102-111.
8. **Guerout-Fleury AM, Frandsen N, Stragier P.** 1996. Plasmids for ectopic integration in Bacillus subtilis. Gene **180:**57-61.
9. **Arnaud M, Chastanet A, Debarbouille M.** 2004. New vector for efficient allelic replacement in naturally nontransformable, low-GC-content, gram-positive bacteria. Appl Environ Microbiol **70:**6887-6891.
10. **Mukherjee S, Yakhnin H, Kysela D, Sokoloski J, Babitzke P, Kearns DB.** 2011. CsrA-FliW interaction governs flagellin homeostasis and a checkpoint on flagellar morphogenesis in *Bacillus subtilis*. Mol Microbiol **82:**447-461.

Figure S1

MR**INHNIAALNTLNRL**SSNNSASQKNMEKLSSGLRINR**AGDDAAGLAISEK**MRGQIRGLEMASK**NSQDGISLIQTA**
EGALTETHAILQRVRELVVQAGNTGTQDKATDLQSIQDEISALTDEIDGISNRTEFNGK**KLLDGTYKVDTATPANQ**
KNLVFQIGANATQQISVNIEDMGADALGIKEADGSIAALHSVNDLDVTKFADNAADTADIGFDAQLKVVDEAINQV
SSQRAK**LGAVQNRLEHTINNLSASGENLTAAESR**IRDVDMAKEMSEFTK**NNILSQASQAMLAQANQQPQNVLQLLR**

Figure S2.

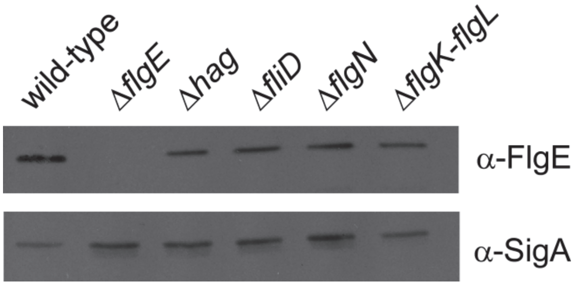

Figure S3

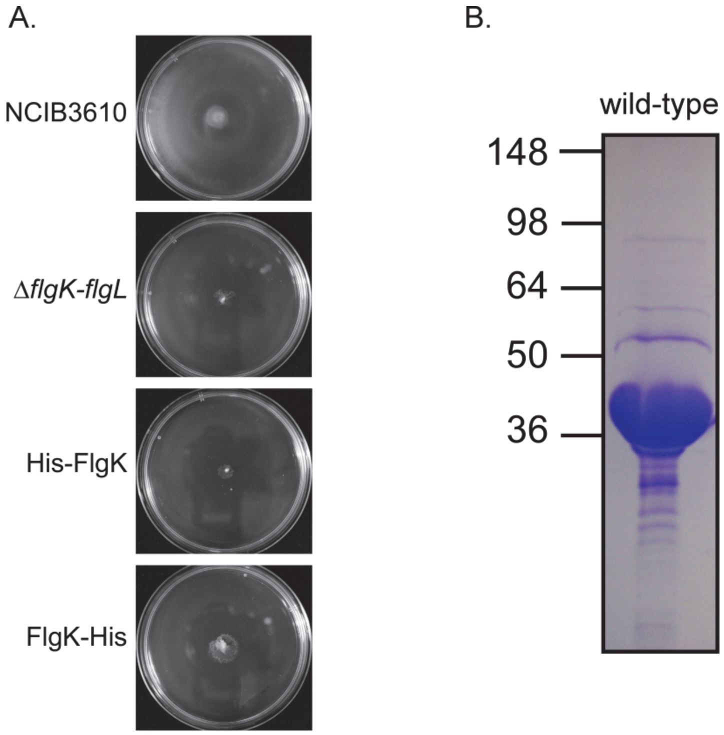
A.
NCIB3610
ΔflgK-flgL
His-FlgK
FlgK-His
B.
wild-type
148
98
64
50
36
